# Signatures of muscle disuse in spaceflight and bed rest revealed by single muscle fiber proteomics

**DOI:** 10.1093/pnasnexus/pgac086

**Published:** 2022-06-11

**Authors:** Marta Murgia, Stefano Ciciliot, Nagarjuna Nagaraj, Carlo Reggiani, Stefano Schiaffino, Martino V Franchi, Rado Pišot, Boštjan Šimunič, Luana Toniolo, Bert Blaauw, Marco Sandri, Gianni Biolo, Martin Flück, Marco V Narici, Matthias Mann

**Affiliations:** Department of Biomedical Sciences, University of Padova, Via Ugo Bassi, 58/B, 35131 Padua, Italy; Max-Planck-Institute of Biochemistry, Am Klopferspitz 18, 82152 Martinsried, Germany; Veneto Institute of Molecular Medicine, Via Orus 2, 35129 Padua, Italy; Department of Molecular Medicine, University of Pavia, Via Forlanini 6, 27100 Pavia, Italy; Bruker Daltonik, GmbH, Fahrenheitstr. 4, 28359 Bremen, Germany; Department of Biomedical Sciences, University of Padova, Via Ugo Bassi, 58/B, 35131 Padua, Italy; Science and Research Center Koper, Institute for Kinesiology Research, Garibaldijeva Street 1, 6000 Koper, Slovenia; Veneto Institute of Molecular Medicine, Via Orus 2, 35129 Padua, Italy; Department of Biomedical Sciences, University of Padova, Via Ugo Bassi, 58/B, 35131 Padua, Italy; Science and Research Center Koper, Institute for Kinesiology Research, Garibaldijeva Street 1, 6000 Koper, Slovenia; Science and Research Center Koper, Institute for Kinesiology Research, Garibaldijeva Street 1, 6000 Koper, Slovenia; Department of Biomedical Sciences, University of Padova, Via Ugo Bassi, 58/B, 35131 Padua, Italy; Department of Biomedical Sciences, University of Padova, Via Ugo Bassi, 58/B, 35131 Padua, Italy; Veneto Institute of Molecular Medicine, Via Orus 2, 35129 Padua, Italy; Department of Biomedical Sciences, University of Padova, Via Ugo Bassi, 58/B, 35131 Padua, Italy; Veneto Institute of Molecular Medicine, Via Orus 2, 35129 Padua, Italy; Clinical Department of Medical, Surgical and Health Sciences, Strada di Fiume 447, 34149 Trieste, Italy; Department of Medicine, University of Fribourg, Chemin du Musee 5, 1700 Fribourg, Switzerland; Department of Biomedical Sciences, University of Padova, Via Ugo Bassi, 58/B, 35131 Padua, Italy; Science and Research Center Koper, Institute for Kinesiology Research, Garibaldijeva Street 1, 6000 Koper, Slovenia; CIR-MYO Myology Center, Viale G Colombo 3, 35121 Padua, Italy; Max-Planck-Institute of Biochemistry, Am Klopferspitz 18, 82152 Martinsried, Germany; NNF Center for Protein Research, Faculty of Health Sciences, University of Copenhagen, Blegdamsvej 3B, Building 6.1, 2200 Copenhagen, Denmark

**Keywords:** proteomics, skeletal muscle, single fibers, bed rest, astronauts

## Abstract

Astronauts experience dramatic loss of muscle mass, decreased strength, and insulin resistance, despite performing daily intense physical exercise that would lead to muscle growth on Earth. Partially mimicking spaceflight, prolonged bed rest causes muscle atrophy, loss of force, and glucose intolerance. To unravel the underlying mechanisms, we employed highly sensitive single fiber proteomics to detail the molecular remodeling caused by unloading and inactivity during bed rest and changes of the muscle proteome of astronauts before and after a mission on the International Space Station. Muscle focal adhesions, involved in fiber–matrix interaction and insulin receptor stabilization, are prominently downregulated in both bed rest and spaceflight and restored upon reloading. Pathways of antioxidant response increased strongly in slow but not in fast muscle fibers. Unloading alone upregulated markers of neuromuscular damage and the pathway controlling EIF5A hypusination. These proteomic signatures of mechanical unloading in muscle fiber subtypes contribute to disentangle the effect of microgravity from the pleiotropic challenges of spaceflight.

Significance Statement In the absence of mechanical loading, skeletal muscle undergoes atrophy with loss of strength and detrimental metabolic effects. We use mass spectrometry-based proteomics to compare single skeletal muscle fibers of healthy volunteers before and after 10 days of continuous bed rest. In these multinucleated single cells, we find that protein complexes responsible for the interaction with the extracellular matrix and for force transmission are strongly downregulated in the unloading phase. Slow and fast fiber types undergo partially different changes. Our parallel proteomic analysis of muscle biopsies of astronauts before and after a 6-month mission on the International Space Station highlights similar changes caused by lack of gravity despite daily exercise. We lay a molecular basis for countermeasures.

## Introduction

Human muscles are composed of three types of multinucleated single cells, the muscle fibers, named slow-type 1, fast-2A, and fast-2X, with distinct contractile performance and metabolic profile ranging from slow/oxidative to fast/glycolytic. The expression of one specific isoform of myosin heavy chain (MYH) defines fiber type and is the main determinant of contractile properties. In addition, some fibers are mixed-type, expressing two MYH isoforms, mostly 1+2A and 2A + 2X, at comparable levels (1).

In healthy humans, 2 days of immobilization are sufficient to trigger a loss of muscle mass of about 2% progressively increasing to 17% over the course of 8 weeks (2, 3). The concomitant loss of muscle strength is proportionally greater than the actual decrease in muscle mass (4–6). At the cellular level, mechanical unloading causes marked alterations in single fiber morphological, histochemical, and mechanical properties (7–10). Muscle unloading or inactivity are accompanied by glucose intolerance and other detrimental effects on whole-body metabolism (11, 12).

At Earth gravity, these perturbations are fully reverted by physical activity (13). During spaceflight, astronauts experience consistent loss of muscle mass and strength despite daily sessions of intense exercise (5, 14, 15). Muscle atrophy is caused by the absence of gravitational loading and imposes an additional burden to the health of astronauts (16–18) on top of other challenges inherent to spaceflight, including ionizing radiation and disruption of circadian rhythms (19, 20).

Earth-bound models of disuse, such as bed rest, unilateral lower limb suspension, or dry immersion, can dissect the effects of muscle unloading from the other environmental changes inherent to spaceflight (21, 22). These simulated microgravity models allow the sequential analysis of structural and functional changes at multiple time points, which has so far proved impossible during spaceflights. In addition, rodents (23) and cell lines (24) subjected to spaceflight or parabolic flight are contributing an increasing body of mechanistic information.

With the perspective of human missions in deep space drawing near, mechanistic knowledge of the effects skeletal muscle unloading is essential for the development of countermeasures. Slow fibers, performing tasks of posture and tonic activity, and fast fibers, engaged in phasic movements requiring more force, have been shown to respond differently to disuse (18). The optimization of targeted countermeasures decelerating atrophy in astronauts would benefit from fiber type-resolved mechanistic knowledge of unloading-mediated atrophy. We previously applied mass spectrometry (MS)-based proteomics to single muscle fibers for the first time (25) and used this approach to detail the specific response of each fiber type to activity (26), and aging (27). In addition, this allowed us to reveal muscle fiber-intrinsic changes devoid of the contribution of other tissue components like blood vessels and interstitial cells. For muscle inactivity specifically, fibro-adipogenic progenitors and intramuscular fat infiltration might significantly alter the cell composition of whole muscle lysate (28).

Here, we analyzed 233 muscle fibers isolated from the biopsies of young healthy volunteers who underwent 10 days of continuous bed rest without any muscle loading or physical activity. In parallel, we measured the muscle proteome of two astronauts, from biopsies taken before and after 6 months of permanence on the International Space Station (ISS) with daily exercise as countermeasure for unloading. We applied state-of-the-art trapped ion mobility spectrometry (TIMS) time of flight (TOF) quantitative MS-based proteomics, which has recently led to the first measurement of a mononucleated single cell proteome (29).

We compare the remodeling of skeletal muscle caused by spaceflight and bed rest on Earth using the same proteomic workflow and quantification methods. Our study reveals proteomic signatures controlled by weight load in muscle, possibly contributing to insulin signaling and glucose tolerance. Our dataset is a resource to disentangle the direct effects of unloading and disuse of skeletal muscle from the consequences of other complex challenges of microgravity, such as changes in neural adaptations, altered muscle perfusion due to cardiovascular deconditioning, osteoarticular modifications and cosmic radiation (30–32).

## Results

### Proteomic workflow and single muscle fiber type assignment

**Fig. 1.**
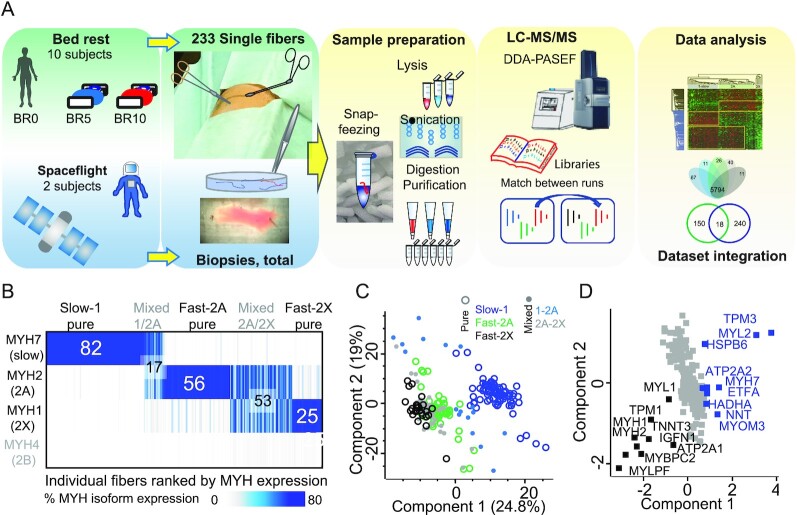
Proteomic workflow, MS-based muscle fiber type assignment and phenotype. (A) MS-based proteomics workflow for the preparation and analysis of single muscle fibers from bed rest volunteers, whole lysates from astronauts, and samples for library generation. For details see main text and Materials and Methods. (B) Fiber-type assignment based on the % expression of four adult isoforms of MYH in 233 single muscle fibers. Fibers of each type were ranked by percentage purity. Both pure and mixed fibers are shown in the heatmap. The total number of pure and mixed fibers of each type in the dataset is indicated. Each vertical line represents a single fiber. Slow-1 fibers were defined as pure when expressing > 80% of MYH, 2A, and 2X when expressing > 70% of MYH2 and MYH1, respectively. The other fibers were defined as mixed-type. The adult isoform MYH4 (gray font) is only present in trace amount in human limb muscles. (C) Principal component analysis (PCA) of pure fibers (circles) and mixed-type fibers (dots), using only proteins expressed in all fibers. *N* = 233 pure and mixed-type single fibers, 178 proteins. (D) PCA loadings, with proteins driving the separation of slow-1 fibers in blue and of fast 2A and 2X in black. Proteins annotated as mitochondrial are significantly enriched in slow fibers (*P* = 1E-15). *N* = 233 single fibers, 178 proteins.

We dissected and analyzed by MS a total of 233 single muscle fibers from ten volunteers undergoing strict bed rest for 10 days (Fig. 1A, Table S1, Supplementary Material). Muscle biopsies were taken immediately before bed rest (BR0), at days 5 (BR5) and 10 (BR10). We then examined whole muscle lysates of two astronauts who spent 6 months on the ISS, undergoing one muscle biopsy before and two after the mission. Our proteomic analysis quantified over 7,500 proteins in the muscle fibers (2,900 per single fiber on average) and 7,100 in the whole muscle lysates of astronauts (Tables S2, S3, and Figure S1A, Supplementary Material). Over 93% of proteins were quantified at all three time points of bed rest and 65% in all 10 donors; in astronauts, 74% of proteins was quantified at three time points and 82% in both individuals (Figure S1B, Supplementary Material). This confirms the robustness and stability of our workflow and allows comprehensive systematic comparisons within and between our datasets.

Based on MS analysis of MYH isoforms (see Methods), we assigned 82 fiber types as pure slow-1, 56 as fast-2A, and 25 as fast 2X. Fibers expressing less than the threshold % of a single MYH isoform were defined as “mixed fibers” and assigned to the 1/2A and 2A/2X group according to the more abundant isoforms (Fig. 1B). Other MYH isoforms were expressed with MS-intensities three orders of magnitude lower than the majority isoforms (Table S4, Supplementary Material). The proportion of fiber types collected at the three time points of bed rest showed only minor variations (Figure S1C, Supplementary Material). Comparing the proteomes of slow and fast fiber types highlighted 470 proteins with significantly different expression between pure slow-1 and fast-2A fiber and 481 between Slow-1 and fast-2X (Figure S1D, S1E, Tables S5, and S6, Supplementary Material). Myosin and troponin isoforms displayed the largest and most significant differences between fiber types, providing positive control.

Principal component analysis (PCA) yielded a clear separation of slow-1 fibers from the fast-types along component one. Fast-2A and 2X showed some overlap. Interestingly, fibers assigned as mixed type distributed between the pure groups expected by their MYH composition (Fig. 1C). The PCA loadings indicate that many mitochondrial proteins and slow muscle protein variants, such as the ER calcium ATPase ATP2A2, drive the separation of slow from fast fibers. The latter express typical fast contractile proteins and the ATP1A1 isoform (Fig. 1D). Unsupervised hierarchical clustering on the proteins with significantly different expression between at least two of the three fiber types (ANOVA) yielded a vertical separation into fiber types (with only three exceptions, see arrows). In addition, it showed clusters with significant annotation enrichments in fast structural proteins (myosin II complex) and proteins involved in glycogen metabolism limited to fast 2A and 2X fibers. Conversely, slow contractile proteins (termed cardiac, as many slow muscle isoforms are also expressed in cardiac muscles) as well as mitochondrial protein were enriched in slow-1 fibers (Figure S1F, Supplementary Material). The latter cluster is shared with fast-2A fibers as expected from the rather high mitochondrial content characterizing this fiber type in humans (33).

Pearson's correlation coefficients of all single fibers per individual (median expression), ranged from 0.82 to 0.90 for the least to the most similar subjects. For fiber types, the average Pearson's correlation between slow-1 and fast-2X fibers was 0.79 and was thus lower than the correlation among different donors (average 0.86). Thus, at the proteome level, differences among fiber types outweigh those between subjects. The two astronauts had comparably low Pearson correlation, indicating high variability and different activity load between the two subjects (Figure S1G, Supplementary Material) (5). Our proteomic quantification of muscle fibers and whole muscle lysates resulted in over 95% coverage of specific structural features of the skeletal muscle such as contractile proteins and sarcoplasmic reticulum as well as over 90% of broader annotations such as respiratory chain, ribosome, and proteasome (Figure S1H, Supplementary Material).

### Costameric proteins are prominently regulated by bed rest-induced unloading in muscle fibers

We carried out ANOVA comparing the proteomes of single fibers isolated at BR0, BR5, and BR10, to retrieve the proteins with significantly different expression (*P* < 0.05) between at least two time points (Table S7, Supplementary Material). After excluding typical plasma protein annotations (likely the result of blood traces and coisolation of segments of blood capillaries on fiber surface), proteins downregulated during bed rest were uniquely enriched in pathways of cell adhesion and interaction with the extracellular matrix. This finding indicates that the tight interaction of fibers and matrix, a fundamental relay between fiber contraction and movement, are actively maintained by weight loading and decrease in its absence. Categories “actomyosin,” “Z disk,” and “calcium channel” were also significantly decreased during bed rest, suggesting that loss of muscle mass measured during bed rest is accompanied by remodeling at the level of sarcomeres and excitation–contraction coupling (Figure S2A, Supplementary Material).

**Fig. 2.**
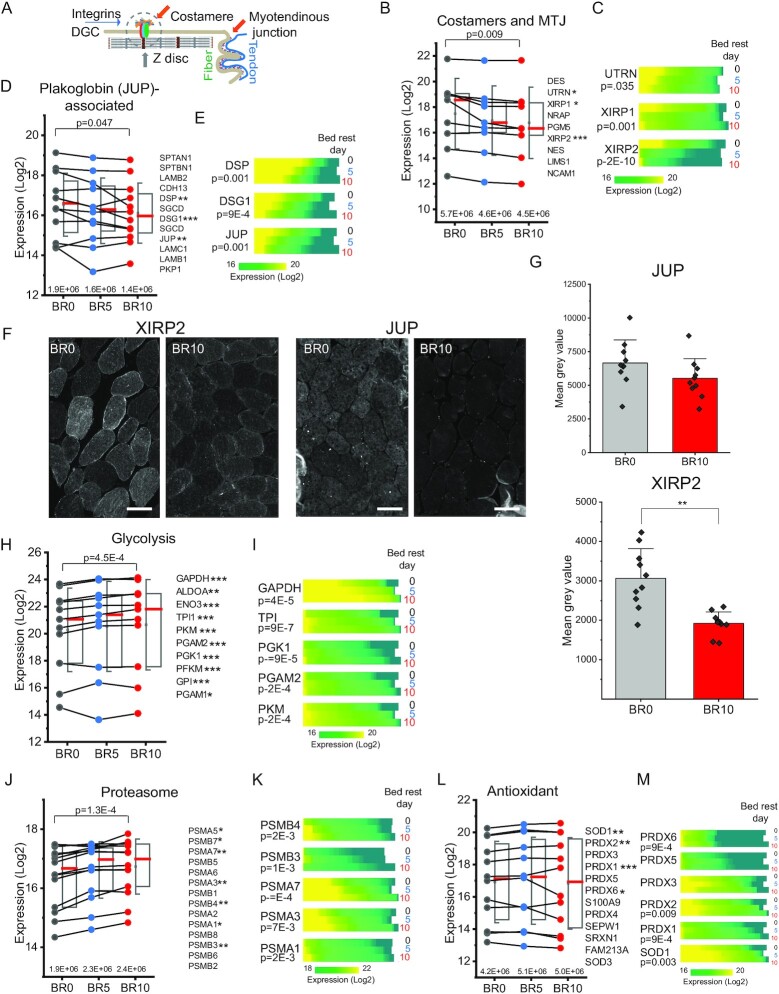
Time-dependent proteomics features of muscle fiber disuse and unloading. (A) Schematic representation of costameres and MTJ involved in muscle fiber- and muscle fiber-tendon adhesion. Some proteins mentioned in the text, such as XIRP1, XIRP2, and NRAP are located both at costameres and MTJ. (B) Trendlines with half boxplots of the expression of proteins enriched at the costameres and MTJ, with overall statistical significance on top (BR10 vs. BR0, paired T-test). Box shows median in red, 75th and 25th percentile, whiskers SD. The protein list on the right is ordered by decreasing expression at BR10. Proteins with statistically significant expression difference in fibers before bed rest (BR0) and at BR10 are marked with asterisks (*P < 0.05, ** *P* < 0.01, and *** *P* < 0.0001). T-test, *N* = 78 fibers at BR0 and 80 at BR10. Each fiber corresponds to one raw file and is considered as an independent experiment. The summed intensity of the proteins at each time point is shown above the *x-*axis. (C) Heatmap of the expression in all fibers of three MTJ proteins with significant decrease upon unloading, UTRN, XIRP1, and XIRP2. Protein expression (LFQ intensity, Log2, color scale at bottom) is split into horizontal blocks, showing all fibers at each time of bed rest, as indicated on the right. BR0, 78 fibers, BR5 75, and BR10 80. *P-*value (T-test, BR10 vs. BR0) is reported on the left. (D) Trendlines with half boxplots of the expression of proteins enriched at the costameres in complex with plakoglobin (JUP). See panel (B). (E) Heatmap showing the expression in all fibers of three costameric proteins with significant decrease upon unloading (see panel C), DSP, DSG, and JUP. See panel (C). (F) Immunofluorescence analysis comparing the expression of XIRP2 and JUP at BR0 and BR10 in muscle biopsies of bed rest subjects. Representative images of the staining at BR0 and BR10 of the same subject. Brightness was digitally enhanced by an identical factor for paired images (XIRP2 300, XIRP1 150, and JUP 100). (G) Bar graphs of median gray value in the stained biopsy of all subjects (*N* = 10, > 50 fibers/subject, T-test). (H) Trendlines with half boxplots of the expression of glycolytic enzymes. See panel (B). (I) Heatmap showing the expression in all fibers of five glycolytic enzymes with significant increase in expression upon unloading. See panel (C). (J) Trendlines with half boxplots of the expression of proteasome proteins. See panel (B). (K) Heatmap showing the expression in all fibers of five proteasome subunits with significant increase in expression upon unloading See panel (C). (L) Trendlines with half boxplots of the expression of proteins involved in protection from reactive oxygen species (keywords annotation antioxidant). Description as in (A). (M) Heatmap showing the expression in all fibers of six antioxidant enzymes with significant increase in expression upon unloading. Description as in (B).

Force transmission in muscle fibers is based on the structural connection between sarcomeres and the extracellular matrix and mediated by several protein complexes. The two main anchoring structures involved in force transmission are the myotendinous junction (MTJ) and costameres (34). The former connects each fiber to a segment of the tendon, the latter harbors a multitude of functionally cooperating protein complexes connecting the Z disk of the sarcomere to the sarcolemma and further to matrix proteins (schematically represented in Fig. 2A). From BR0 to BR10, we observed a decrease in the median expression of several components of the dystroglycan complex (DGC), the main transmembrane anchoring structure of the costameres connected to dystrophin on the cytosolic side (35) (Figure S2B, Supplementary Material). The expression of key proteins involved in fiber–matrix adhesion, associated both with costameres and with the MTJ, was also significantly downregulated during bed rest (Fig. 2B). In total, based on summed expression, costamere and MTJ protein abundance decreased 20% over 10 days of unloading (Fig. 2B above *x-*axis). We ranked the expression of the most significantly regulated proteins, namely Xin actin-binding repeat-containing proteins 1 and 2 (XIRP1 and XIRP2) and utrophin (UTN), at each BR time in a heat map (BR0, *N* = 78, BR5, *N* = 75, and BR10 *N* = 80). We observed a progressive shift towards lower intensities from BR0 to BR10, indicating that more fibers expressed these proteins at lower levels in the unloading phase (Fig. 2C). Plakoglobin/gamma catenin (JUP) is a component of desmosomes and adherens junctions, interacting with desmoglein (DSG1) and desmoplakin (DSP) in many cell types. In skeletal muscle, JUP is the key component of a newly discovered sarcolemmal signaling complex interacting with the DGC at the costameres (36, 37). We observed a consistent decrease in the expression of several members of this complex in response to bed rest. The summed intensity of the complex was reduced by 25% after 10 days of unloading (Fig. 2D). JUP, DSP, and DSG1 also showed highly significant decreases during bed rest, as an effect of many individual fibers shifting towards lower expression over time (Fig. 2E).

We validated the downregulation of the complexes involved in fiber–matrix interaction and force transmission by immunofluorescence with antibodies specific for XIRP2 and JUP on cross-sections of muscle biopsies of all ten volunteers involved in the study. (Fig. 2F and G). Comparing section of biopsies from BR0 with those of BR10 by quantitative image analysis, confirmed a significant decrease (*P* < 0.05) in the expression of XIRP2. JUP expression tended to decrease, but the variability among donors was higher (Fig. 2G). XIRP1 also showed significant downregulation comparing BR0 and BR10 (Figure S2C and S2D, Supplementary Material).

The proteins whose expression increased during bed rest were most significantly enriched in annotations related to protein turnover (proteasome, elongation factor, chaperone, and hypusine), antioxidant, and stress response as well as carbohydrate metabolism, the latter comprised in the term “glycolysis” (Figure S2E, Supplementary Material). Indeed, the enzymes involved in the core reactions of glycolysis increased in abundance during bed rest (Fig. 2H and I), similarly to proteasomal proteins (Fig. 2J and K) and antioxidant enzymes (Fig. 2L and M). Translation initiation and elongation factors were also enriched among the proteins increasing their expression at BR10 compared to BR0, though the trend was variable for different members of this functional group (Figure S2F and S2G, Supplementary Material). Interestingly, the translation initiation/elongation factor EIF5A, the only mammalian protein containing the modified aminoacid hypusine (38) was significantly upregulated during bed rest, as indicated by the over 70-fold enrichment in this annotation (see Figure S2E, Supplementary Material). In addition, we found that the enzymes specifically catalyzing EIF5A hypusination at amino acid residue lys50, deoxyhypusine synthetase (DOHS), and deoxyhypusine hydroxylase (DOHH) were also concomitantly upregulated upon unloading (Figure S2H, Supplementary Material).

### Fiber type-specific and fiber type independent proteomic changes induced by bed rest

Proteomic studies have shown that slow and fast fiber types undergo different changes in response to training (26) and during aging (27, 39). To explore the effects of unloading on the proteome of individual fiber types, we carried out ANOVA between the three times of bed rest comparing only the pure fibers of each type. We then retrieved the proteins that were significantly up- and downregulated in the course of bed rest in each fiber type.

**Fig. 3.**
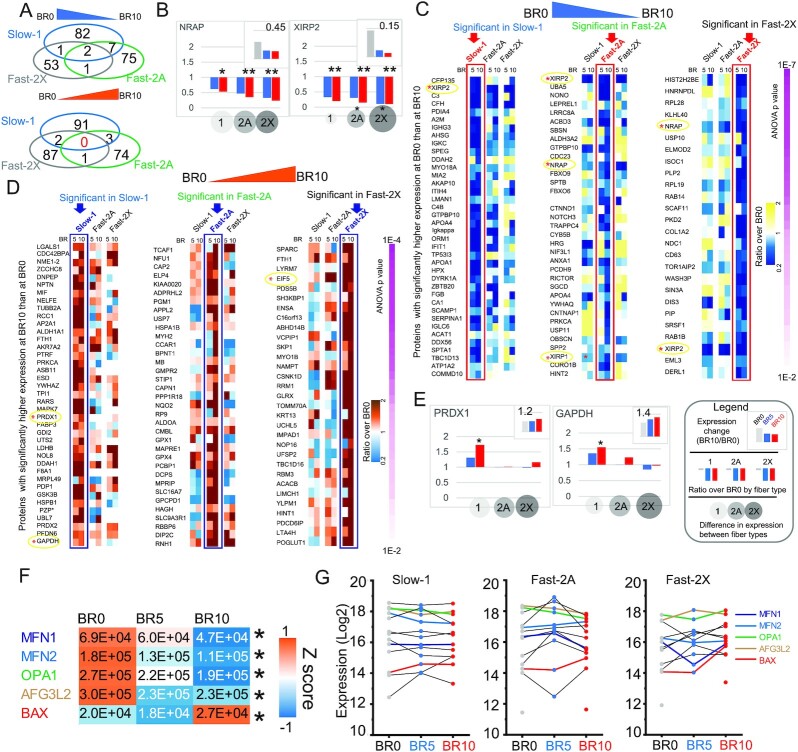
Fiber type-specific effects of muscle unloading. (A) Proteins with significantly lower (top) and higher (bottom) expression at BR10 compared to BR0. (B) Fiber type-resolved effects of muscle unloading on the two proteins significantly downregulated at BR10 in all three fiber types. The ratio between expression at BR10 and BR0 in all fibers (top graph), the ratio in individual pure fiber types (middle graph) and the relative expression in different fiber type (bottom circles) is shown as depicted in the legend next to panel (E). BR0, gray bars; BR5, blue bars; and BR10, red bars. T-test (*P < 0.05). (C) Heatmap showing the expression ratio (BR10/BR0) of proteins with expression BR0 > BR10 according to the color scale shown on the right. Only proteins with *P*-value < 0.01 (ANOVA and post hoc tests comparing only pure fibers of the same type) are shown in the heatmaps, the remaining proteins with 001 < *P-*value < 0.05 are listed in Table S8 (Supplementary Material). The proteins are ranked by *P-*value, as indicated on the right. For each fiber type, marked on top, the corresponding column in which the expression change is statistically significant is marked by a rectangle. (D) Heatmap showing the expression ratio (BR10/BR0) of proteins with expression BR0 < BR10 according to the color scale shown on the right. See panel (C) and Table S8 (Supplementary Material). (E) Fiber type-resolved effects of muscle unloading of two proteins upregulated by unloading only in type1-slow fibers. The ratio between expression at BR10 and BR0 in all fibers (top graph), the ratio in individual pure fiber types (middle graph), and the relative expression in different fiber type (bottom circles) is shown as depicted in the legend. BR0, gray bars; BR5, blue bars; and BR10, red bars. T-test (*P < 0.05). See detailed legend in panel (E). (F) Heatmap with expression (LFQ (explain) intensity) of five proteins involved in mitochondrial network remodeling, with significantly different expression between BR0 and BR10 (marked by asterisk on the right). Color scale, Z score as indicated. BR0 *N* = 78, BR5 *N* = 75, and BR10 N-80. (G) Profile plots by fiber type of the expression of protein annotated as “mitochondrial fusion” and “fission” (GOBP). The protein list, ordered by expression at day 10 in decreasing order, is shown on the right. The profiles and names of the five proteins with significant expression are labeled in colors as shown on the right.

Fiber types displayed unique sets of proteins significantly regulated during bed rest, with only two proteins commonly downregulated and none commonly upregulated in all three fiber types (Fig. 3A). The former two were the Z disk protein Nebulin-related-anchoring protein (NRAP) and XIRP2 whose expression ratio BR10/BR0 decreased more for the fast fiber types 2A and 2X. (Fig. 3B; Table S8, Supplementary Material). We then visualized the expression ratio BR10/BR0 of the most significantly downregulated proteins (*P* < 0.01) in slow-1, fast-2A and fast-2X fiber types, respectively (Fig. 3C; Table S8, Supplementary Material). We observed that proteins upregulated at BR10 in slow-1 fibers were uniquely enriched in annotations related to ROS detoxification and carbohydrate metabolism (Fig. 3D; Figure S3A, Supplementary Material). Indeed, peroxiredoxin 1 (PRDX1) and glyceraldehyde 3-phosphate dehydrogenase (GAPDH) were upregulated only in slow-1 fibers during bed rest (Fig. 3E), together with other ROS detoxifying enzymes (Figure S3B, Supplementary Material). Some proteasomal subunits, like PSMA3 and 7, which showed increased expression in slow-1 and fast-2A but not in fast-2X fibers (Figure S3C, Supplementary Material).

We asked whether the observed fiber type-specific increase in detoxifying enzymes could reflect fiber type-specific changes in expression or composition of the five respiratory chain complexes, which are the main source of ROS. To this end we calculated the median expression of each complex (schematically represented in Figure S3D, Supplementary Material) in muscle fibers of the same type at the three BR time points and detected small-sized expression changes comparing fibers isolated prebed rest with those isolated after 5 and 10 days of unloading. All five complexes showed a transient increase at BR5 in slow-1 fibers, which reverted to prebed rest levels at BR10. (Figure S3D, Supplementary Material). The subtle but consistent changes in the expression of RC complexes might be linked to variations in shape and connection of the mitochondrial network, which accompanies muscle plasticity and takes part in the regulation of muscle mass (40, 41). We analyzed the expression of main players of mitochondrial fission and fusion, which regulate the architecture of the mitochondrial network. A total of three proteins involved in mitochondrial fusion (OPA1, MFN1, and MFN2) and the protease AFG3L2 showed significantly lower expression (*P* < 0.05) in muscle fibers at BR10 compared with BR0. Conversely, the expression of BAX, a proapoptotic protein which associates with the outer membrane when mitochondria undergo fission, increased significantly at BR10 (Fig. 3F). These and other proteins controlling the architecture of the mitochondrial network had fiber type-specific expression changes in response to unloading (Fig. 3G). This might be linked to a different response to atrophy-inducing signals of slow and fast fibers observed in various pathophysiological conditions (42).

As mentioned above, loss of muscle mass measured during bed rest in this cohort (9) is accompanied by remodeling at the level of sarcomeres and excitation–contraction coupling (see Figure S3, Supplementary Material). As sarcomeric and EC coupling proteins are highly fiber type-specific, we calculated their median expression in all pure and mixed fiber types separately. We then calculated the fold change over BR0 of the median expression of all single fibers isolated at BR5 and BR10. We observed few significant changes for myosins, possibly due to their long half-life (43) and troponins as well as for intermediate filaments and associated proteins and the Z disk (Figure S3E–S3I, Supplementary Material). Comparably more proteins of the sarcoplasmic reticulum and T-tubule involved in EC coupling had significant expression changes during bed rest (Figure S3J, Supplementary Material), suggesting that unloading is associated with structural rearrangements of the SR, which might contribute to the decreased performance measured at whole muscle level in this bed rest cohort (9).

### Signatures of muscle unloading from spaceflight and bed rest

**Fig. 4.**
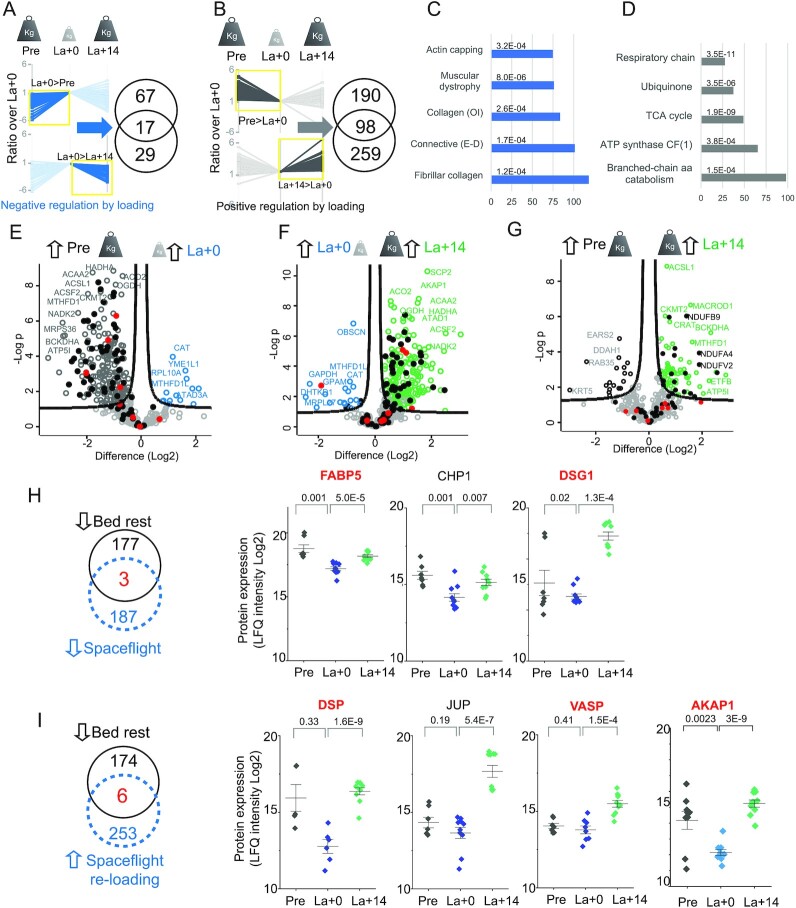
Effects of spaceflight on the whole muscle and mitochondrial proteome. (A) Left, expression ratio (Log2) of proteins negatively regulated by weight load, with higher expression at La + 0 compared to preflight (ANOVA, significant in both astronauts). The darker line color and yellow box indicate the side with statistically significant expression difference (ANOVA and Tukey's HSD, *N* = 5). Bottom, the same analysis for proteins decreasing their expression upon reloading on Earth. Weight load changes during the phases of the mission are schematically represented on top. Right, number of proteins significantly up-and downregulated shown in the corresponding profile plots, intersection shows the number of proteins with significant, opposite expression changes in both phases. (B) Same analysis for proteins positively regulated by weight load. (C) Annotation enrichments among proteins negatively regulated by weight load (intersection of Venn diagram in panel A, *N* = 17 proteins). (D) Annotation enrichments among proteins positively regulated by weight load (intersection of Venn diagram in panel B, *N* = 98 proteins). OI, osteogenesis imperfecta. E–D, Ehlers–Dannlos syndrome. (E) Comparison of the muscle mitochondrial proteome (Mitocarta 2) of astronauts from the biopsy before mission (Pre, Earth gravity, represented by large weighing weight) and on the day of landing after 6 months on the ISS (La + 0, microgravity, represented by small weighing weight). A subset of proteins with large expression difference in the two conditions is labeled with their gene name. Respiratory chain proteins are marked by filled dots. Red dots, encoded by mtDNA. (F) Comparison of the muscle mitochondrial proteome from the biopsy before mission with that taken after 14 days of normal daily activity on Earth postflight (La + 14, see also E). (G) Comparison of the muscle mitochondrial proteome from the biopsy on the day of landing with that taken after 14 days of normal daily activity on Earth postflight (see E and F). (H) Left, number of proteins with significant downregulation after bed rest (black) and after space flight (blue dashed), with three common proteins. Proteins lists derive from ANOVA (Bed rest, *N* = 83 and 80 single fibers. Astronauts, *N* = 10, 2 subjects, five technical replicates). Right, expression of the three common proteins in skeletal muscle before (Pre), on the day of landing (La + 0) and 14 days after landing (La + 14). *N* = 10, 2 subjects, five technical replicates. *, *P* < 0.05, **, *P* < 0.01, T-test. Protein name in red indicates common to panel I. (I) Left, number of proteins with significant downregulation after bed rest (left, red filled dots) and upregulated in astronauts after 14 days of normal daily activity on Earth postflight (right, blue filled squares), with six common proteins. Right, expression of four of the six common proteins, the other two are in panel H (name in red). See panel H. (G) Column scatter detailing the expression of JUP and its interactor DSG1/desmoglein and DSP/desmoplakin in skeletal muscle before (Pre), on the day of landing (La + 0) and 14 days after landing (La + 14). *N* = 10, two subjects, five technical replicates. *, *P* < 0.05, **, *P* < 0.01, T-test. (H) Proteins with significant upregulation after bed rest (left, red filled dots) and downregulated in astronauts after 14 days of normal daily activity on Earth postflight (right, blue filled squares). See panel (E).

Spaceflight poses complex challenges to the human body, which are only partially reproducible at Earth gravity. We asked which proteomic changes of muscle fibers during unloading on Earth could recapitulate the behavior of muscle in microgravity. To this aim, we quantified over 7,000 proteins from whole muscle lysates of two crew members of a 6 months ISS mission. We measured technical quintuplicates of muscle biopsies taken 75 days before flight (Pre), 1 day after landing (La + 0), and during muscle recovery after 14 days of reloading on Earth (La + 14). MYH composition measured by MS in the soleus biopsies showed a large majority of slow fibers expressing MYH7, with minor fluctuations at the three phases of the mission (Figure S4A, Supplementary Material). This indicates negligible fiber type changes, possibly due to the due to the intensive training which was adopted as a countermeasure on the ISS. We then carried out ANOVA separately for each astronaut and performed subsequent analyses only on proteins that changed in expression significantly and with the same trend in both astronauts (Table S9, Supplementary Material). Based on the expression ratio over the day of landing, we observed that the proteins with significant expression changes had an opposite trend between the mission phases, following the switch of loading conditions. This indicates that the expression of these proteins is directly or indirectly regulated by weight load, either negatively (Fig. 4A) or positively (Fig. 4B).

Within the proteins significantly upregulated after spaceflight and downregulated during reloading on Earth, we observed enrichments in annotations of the extracellular matrix, including collagens and laminin (Fig. 4C) suggesting a link between muscle loading and the control of matrix composition (44). It is possible that muscle fiber atrophy changes the ratio between contractile tissue and extracellular matrix without changing the absolute amount of connective tissue, as shown recently in a study of 60 days bed rest (45). The relative increase in matrix components measured by MS in the biopsies postflight likely correlates with structural remodeling. Indeed, extensive changes in muscle morphology correlating with atrophy were previously shown in these two astronauts using transversal magnetic resonance and ultrasonography (5). The proteins positively regulated by weight load were specifically enriched in mitochondrial annotations related to energy metabolism (Fig. 4D).

We thus compared the mitochondrial proteome of astronauts at different times of the mission under different loading conditions. Over 90% of mitochondrial proteins (225 of 238 significant) were expressed at significantly lower levels pre-mission than on the day of landing, corresponding to a 35% loss of total mitochondrial protein intensity (Fig. 4E; Figure S4B, Supplementary Material). This effect was prominent for the components of the respiratory chain (48 of 71, black dots in Fig. 4E; Table S10, Supplementary Material). This was partially reversed after reloading at Earth gravity (Fig. 4F; Figure S4C, Supplementary Material). Interestingly, only 50% of the subunits encoded by mitochondrial DNA (four of eight, red dots) showed significant downregulation in spaceflight, a finding that is also validated by a previous report (17). The expression of eighteen respiratory chain components was higher 14 days after landing than it was before the flight (Fig. 4G). After 2 weeks at Earth gravity, 25% of the respiratory chain protein intensity lost at R + 0 had been recovered (Figure S4C, Supplementary Material).

A total of three proteins were significantly downregulated during unloading both in bed rest and in the two astronauts in spaceflight, namely fatty acid-binding protein 5 (FABP5), calcineurin B homologous protein (CHP1) and DSG1 (Fig. 4H). A total of six proteins that decreased their expression during bed best were upregulated in astronauts after 14 days of reloading on Earth, two of which were also downregulated during spaceflight (Fig. 4H–I, labeled in red). DSG1 and DSP are physical interactors and part of a functional complex with JUP, which is also significantly upregulated upon muscle reloading in astronauts (Figs 2 and 4I). Our results thus show that the expression of focal adhesion proteins at the costameres is promoted and/or maintained by weightbearing muscle activity. JUP and its associated proteins have been shown to stabilize the insulin receptors on the sarcolemma (37). The amount of membrane insulin receptor in muscle was shown to be rate-limiting for insulin signaling (46). This suggests the possibility of a link between insulin resistance, which affects astronauts during space flights (47) and was measured in our study subjects undergoing bed rest (see Table S1, Supplementary Material), and the decrease of JUP and associated proteins occurring in muscle unloading.

A total of 17 proteins were significantly upregulated during both spaceflight and bed rest, indicating that their expression in skeletal muscle increases under muscle unloading and disuse (Figure S4D, Supplementary Material). They were specifically enriched in extracellular matrix and redox annotations (Figure S4E, Supplementary Material).

We previously showed that 10 days of bed rest caused neuromuscular junction (NMJ) damage, as indicated by the upregulation of neural cell adhesion molecule 1 (NCAM1) and other markers of NMJ damage and proposed that NMJ damage underlies the greater decline in muscle force than in muscle size, which is typically measured after muscle unloading (9). Interestingly, we here observed that NCAM1 expression was significantly increased in the biopsies of astronauts sampled on the day of landing and remained higher after 2 weeks on Earth (Figure S4F, Supplementary Material).

## Discussion

In spaceflight, atrophy is more prominent in slow-1 than in fast fibers and typically affects postural muscles such as soleus (8). Thus, a fiber type-resolved omics approach to inactivity would be instrumental for further clarifying the molecular basis of muscle dysfunction affecting astronauts. Here, we measured the proteome of single muscle fibers from subjects in bed rest to discover fiber type-specific changes, which could not be extricated in a whole muscle lysate. We then measured the muscle lysate of astronauts after a mission on the ISS with the same proteomic platform and workflow. From the integration of the two datasets, it is possible to mine the contribution of specific fiber types to the phenotype of unloaded muscle.

**Fig. 5.**
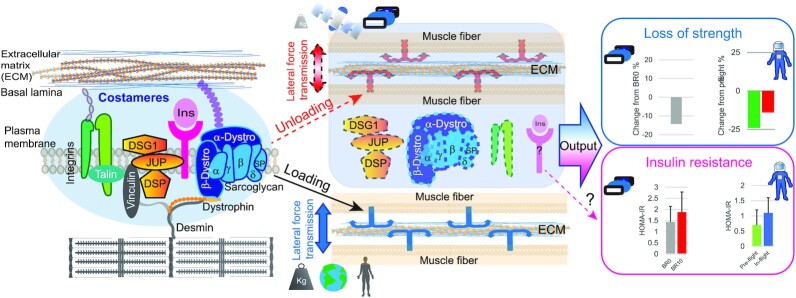
Mechanical load controls the stability of costameric proteins. Both astronauts and volunteers undergoing bed rest experience decreased muscle strength (data derived with permission from Refs (5) and (9)). We hypothesize that unloading, both on Earth and during spaceflight, leads to alteration of lateral force transmission by decreasing the expression of specific proteins located at the costameres (represented as anchors in central panel, see measured expression values in Figs 2 and4; Figures S2, S4, Tables S7, and S9, Supplementary Material), which couple the contractile apparatus to the extracellular matrix. Under normal weight load at Earth gravity, costameres allow for efficient transmission of contractile force from the muscle fibers to the ECM, and they prevent muscle contraction from damaging the sarcolemma. As a consequence of unloading, the expression of specific costameric proteins, thus the transmission of lateral force may result inefficient. Our data also show that the downregulation of plakoglobin (JUP) and its specific interactors DSG1 and DSP is strong both in bed rest and spaceflight. Plakoglobin is part of a complex that stabilizes the insulin receptor (not quantified in this dataset) on the sarcolemma, so it will be of interest to further investigate its role in the development of insulin resistance during muscle inactivity on Earth and in space.

Previous trascriptome and proteome analyses of skeletal muscle during bed rest have shown downregulation of mRNAs annotated to cell adhesion and cytoskeleton (21, 48) and assessed the effects of countermeasures (49, 50). We specifically uncovered a rapid and progressive downregulation of costameric structures, corresponding to the focal adhesions of skeletal muscle, in agreement with previous observations in a model of tendon release in sheep (51). The costameres also accommodate a recently identified signaling hub containing desmosomal components, including plakoglobin (JUP) and the insulin receptor, which activates the PI3K-FOXO signaling preventing muscle atrophy (36). We measured a significant decrease in the expression of JUP, DSG1, and DSP, as well as other interactors of this molecular complex (37) in bed rest. The same proteins were also downregulated after spaceflight in astronauts and their expression was significantly upregulated after 14 days of normal locomotor activity on Earth (schematically represented in Fig. 5).

Compared to the changes observed in transcriptome studies of bed rest, particularly at longer time points, the proteomic changes we measure here are generally smaller in size and limited to a lower number of significant hits (52). Long protein half-lives of contractile proteins (53) may account for part of this difference, as we could detect changes in MYH transcripts in the muscle lysate of these subjects in a previous study (9), which we did not measure here at the proteome level. Interestingly however, the mitochondrial protein changes in astronauts showed an overlapping with the regulated transcripts reported after long spaceflights (17, 19).

Muscle inactivity and unloading are linked to glucose intolerance (54) and both astronauts and bedridden subjects consistently display alterations in insulin sensitivity (47). We propose that JUP and its associated membrane complex, through their stabilizing action on insulin receptors, play a key role in preserving insulin sensitivity under muscle loading conditions. In addition, we detected downregulation of several members of the sarcoglycan complex and the Xin repeat-containing proteins and UTN, located both at the costameres and at the MTJ. Our fiber type-resolved analysis showed that the unloading time-dependent downregulation was generally larger in fast that in slow fibers. In addition, fast fibers tended to have higher basal expression of costameric proteins, and thus contributed most to the bulk downregulation of these adhesion structures. The MTJ is responsible for the tight association between muscle fibers, directly or through the aponeurosis, and the connective tissue of the tendon, and thus relays the contractile force generated in the sarcomere to the locomotor apparatus. In the same cohort of voluntary bed rest, we have previously shown that knee-extensor isometric maximum voluntary contraction decreased by more than 14% in 10 days, while the mid-muscle cross-sectional area of vastus lateralis was reduced by 5.8% (9). We detected damage to the NMJ and proposed that this degenerative process induced by unloading underlies the decrease in muscle force exceeding the loss of muscle mass (9). Here, we measured over 10-fold increase in neural cell adhesion molecule (NCAM), a marker of muscle denervation, in the muscle biopsies of astronauts on the day of landing compared to preflight. This confirms that impaired muscle nerve connection also occurs under microgravity condition and might contribute to the drastic loss of muscle force in astronauts after landing. In addition, we hypothesize that the significant decrease in protein complexes responsible for lateral and longitudinal force transmission, combined with matrix remodeling, could be a further muscle-intrinsic change whereby mechanical unloading causes a decrease in whole muscle contractile force.

The initiation/elongation factor EIF5A was one of several proteins involved in translation, which we found significantly upregulated during bed rest. The expression of translational regulators has recently been shown to not follow the lower translation rate underlying the onset of atrophy in immobilized mouse muscles (55). Active EIF5A contains an unusual spermidine-derived amino acid residue at position 50, hypusine, which is unique in the human proteome and essential for the translational elongation activity. We show that the two enzymes responsible for EIF5A hypusination, deoxyhypusine synthase and DOHH, are also upregulated during bed rest (see Figure S2, Supplementary Material). EIF5A promotes the expression of OXPHOS and TCA proteins in macrophages (56) and its upregulation in unloaded muscle could thus be a compensatory mechanism to preserve oxidative capacity. Alternatively, hypusinated EIF5A might be inducing autophagy, through its ability to regulate the synthesis of transcription factor EB (TFEB) a master regulator of this pathway (57). Further definition of the role of EIF5A in unloading-mediated muscle atrophy might allow to target this unique molecular axis with existing drugs.

Our data show that 10 days of bed rest cause a significant decrease in the abundance of the mitochondrial dynamin like GTPase OPA1, of the mitochondrial outer membrane GTPase mitofusin (MFN1 and MFN2) and of the matrix protease AGF3L2. As these proteins are major regulators of mitochondrial fusion–fission/balance (58), muscle unloading might be linked to variation in shape and connection of the mitochondrial network, which have been shown to accompany pathological muscle remodeling. Indeed, loss of OPA1 causes muscle atrophy in mice (41) and muscle-specific knockout of MFN1 and 2 leads to a significant reduction in exercise performance (59). In addition, downregulation of the mitochondrial AAA protease AFG3L2 has been linked to constitutively active mitochondrial calcium uniporter (MCU) caused by the accumulation of the essential MCU regulator (EMRE) leading to neurotoxicity (60). This mechanism may be involved in the NMJ damage that we previously detected in individuals undergoing bed rest (9). It is tempting to speculate that muscle unloading causes rearrangements of the mitochondrial network by changing the expression of its master regulators.

While 10 days of bed rest caused subtle changes in the mitochondrial proteome, 6-month spaceflight caused a sharp decrease in the expression of the whole mitochondrial proteome, including the respiratory chain, in agreement with previous reports (17, 61). Interestingly, this was not accompanied by major changes in MYH isoforms composition, thus it was not driven by a fiber type shift. As the muscle biopsies of soleus from the two astronauts contained a large majority of slow fibers expressing MYH7 (see Figure S4A, Supplementary Material), most of the changes in the mitochondrial proteome measure here occurred in slow fibers. The different behavior of the mitochondrial proteome during bed rest and spaceflight might be a simple function of time. However, we observed that spaceflight-induced downregulation of the mitochondrial proteome is effectively reverted after 2 weeks of normal reambulation on Earth. Thus, strikingly, large loading-dependent changes in the expression of the respiratory chain occur in a timescale of days. It is conceivable that other challenges of spaceflight different from unloading are also causing OXPHOS defects. Amongst these, we cannot exclude the impact of cosmic radiation on most biological processes and tissue, increasing the risk of damage to DNA, the cell membrane, the cardiovascular system, the nervous and lymphatic systems and stem cells (62, 63). Since the astronauts in our cohort were training daily on the ISS, our data also indicate that the cause-effect relationship between low-medium intensity, long duration exercise (performed on the ISS) and mitochondrial biogenesis (12) is fundamentally altered under microgravity conditions.

In conclusion, our experimental design based on the MS analysis of individual fibers provided single cell-resolved data on muscle-intrinsic proteome changes devoid of the contribution of other cell types, which are found in whole lysate. This feature of the dataset was instrumental to show that plakoglobin and its associated proteins (also expressed in endothelium and blood cells) decreased significantly after spaceflight in the muscle of astronauts. In addition, we could highlight that only slow-1 fibers significantly upregulated peroxiredoxins and superoxide dismutase, an indication that their antioxidant defense is stronger than that of fast fibers. Microgravity-induced loss of muscle strength is greater than the corresponding loss of muscle mass and largely exercise-resistant. Mechanistically, genetic tools that are protective against muscle atrophy on Earth, such as the knockout of the ubiquitin ligase MuRF1 do not rescue muscle mass under microgravity conditions (64). As muscle atrophy leads to spiraling adverse effects for the organism, addressing this conundrum will be crucial for manned missions in deep space.

## Materials and Methods

### Patient cohorts and samples

For the bed rest cohort, single fibers were dissected from muscle biopsies of 10 healthy volunteers (Table S1, Supplementary Material) previously characterized and analyzed (9). Experiments were carried out with approval from the National Ethical Committee of the Slovenian Ministry of Health, with the reference number 0120–304/2019/9 and complied with the guidelines of the 2013 Declaration of Helsinki. The subjects spent 15 consecutive days at the Izola General Hospital (Izola, Slovenia). The protocol included 3 days of familiarization to the study environment and diet. Muscle biopsies were carried out on vastus lateralis muscle prebed rest (BR0, right leg), after 5 days of bed rest (BR5, left leg) and after 10 days of bed rest (BR10, right leg). For the astronaut cohort, two crew members of equal sex and comparable age, were enrolled in the Sarcolab study (5) and tested before and after their half-year mission on the ISS. All procedures were performed in accordance with the guidelines and approved by the Committee for the Protection of Human Subjects (NASA MPA number 7116301606HR, Protocol number 09–3940-Ren-2-Air-1). Muscle biopsies were obtained preflight (days 79 and 76 for the two subjects, respectively), one day after landing (R + 0) and 14 days after landing (R + 14). Written informed consent was obtained from all subjects prior to study inclusion.

### Single fibers sample processing

Fibers were microdissected manually under a stereomicroscope from fresh biopsies kept in an ice-cold solution containing K-propionate (150 mM),  KH2PO4 (5 mM),  Mg Acetate (5 mM),  EGTA (5 mM), and DTT (1 mM). They were individually frozen in dry ice in standard Eppendorf tubes. The fiber isolation was completed within 20 min of the muscle biopsy procedure. Fibers were lysed in 20 µl of LYSE (PreOmics), heated at 95°C for 5 min and sonicated in a water-bath sonicator (Diagenode) for 15 min. with a 50% duty cycle. Proteolytic digestion was carried out by adding to the fiber lysate 500 ng of endoproteinase LysC and 500 ng of trypsin in 10 µl of LYSE buffer. After overnight digestion at 37°C under continuous shaking, the lysate was acidified to a final concentration of 0.1% trifluoro-acetic acid (TFA) and loaded onto StageTip plugs of SDB-RPS. Purified peptides were eluted with 80% acetonitrile-1% ammonia and dried. The dataset consists of 233 single fibers, obtained from 10 subjects at three time points.

### Total muscle lysate sample processing

Muscle lysates of astronauts in RIPA buffer were precipitated in acetone and resuspended in LYSE buffer (PreOmics), heated at 95°C for 5 min and sonicated in a water-bath sonicator (Diagenode) for 15 min. with a 50% duty cycle. Protein concentration in the lysate was adjusted to 2 µg/µl. Proteolytic digestion was carried out by adding 1 µg of endoproteinase LysC and and trypsin per 50 ug of lysate. After overnight digestion at 37°C under continuous shaking, the lysate was acidified to a final concentration of 0.1% TFA and loaded onto StageTip plugs of SDB-RPS. Purified peptides were eluted with 80% acetonitrile-1% ammonia and dried. The dataset consists of technical quintuplicates for the biopsies of two astronauts at three time points.

### Library generation

We generated two peptide libraries, from whole muscle homogenate and human cultured myoblasts and used the MaxQuant match between run function (Fig. 1). To prepare the human muscle library, a fragment of 5 mg of vastus lateralis was crushed in liquid nitrogen and resuspended in 200 µl of LYSE. Human myoblasts were dissolved in LYSE. Both lysates were heated at 95°C for 5 min and sonicated in the water-bath sonicator for 15 min. After measuring protein concentration, 100 ug of each lysate were digested in a total volume of 200 ul of LYSE 5 ug of endoproteinase LysC for at 25°C for 3 h under continuous stirring. The sample was further sonicated and 5 ug of trypsin were added, followed by overnight incubation at 37°C. Peptides were desalted on StageTip plugs of SDB-RPS and eluted into 16 fractions using a Spider Fractionator (65).

### Liquid chromatography and MS

Peptides were separated on 50 cm columns of ReproSil-Pur C18-AQ 1.9 µm resin (Dr. Maisch GmbH) packed in-house. The columns were kept at 60°C using a column oven. Liquid chromatography–mass spectrometry (LC–MS) analysis was carried out on an anEASY-nLC-1200 system (Thermo Fisher Scientific) connected to a TIMS quadrupole time-of-flight mass spectrometer (timsTOF Pro, Bruker Daltonik). A linear 60 min gradient from 5% to 30% B in 47.5 min was applied, followed by an increase to 60% for 2.5 min, by a 5 min wash at 95% buffer B at 300 nl/min and reequilibration for 5 min at 5% buffer B (Buffer A: 0.1% formic acid; buffer B: 0.1% formic acid, and 80% acetonitrile). For ddaPASEF, 1 MS1 survey TIMS-MS and 10 PASEF MS/MS scans were acquired per acquisition cycle. Singly charged precursor ions were excluded with a polygon filter (otof control, Bruker Daltonik GmbH). A dynamic exclusion of 40 s elution was applied.

### Immunofluorescence of muscle sections and image analysis

Muscle biopsies were cut into 10 µm thick cryosections and fixed with 4% PFA for 15 min at RT, then heated in sodium citrate buffer, pH 6.0, at sub-boiling temperature for 40 min in microwave oven (550 W). Sections were then stained with anti-XIRP1 (1:500, HPA016750, rabbit polyclonal IgG, Atlas Antibodies), or anti-XIRP2 (1:50, HPA034813, rabbit polyclonal IgG, Atlas Antibodies), or anti-JUP (1:50, AP33204SU-N, guinea pig polyclonal antibody, Origene) and costained with antidystrophin (1:100, MANDRA1 (7A10), mouse monoclonal, DSHB, Iowa University), in PBS with 1% BSA overnight at 4°C. The sections were stained with Hoechst prior to mounting with a saturated solution of polyvinyl alcohol with 30% glycerol.

16-bit gray scale raw images were acquired with a Leica DM6B microscope, equipped with a DFC 7000T camera, and quantified with FIJI (ImageJ v. 1.53c, NIH). Muscle sections were manually outlined with freehand selection tool and mean gray value for each muscle was measured. Mean gray value was also recorded in regions adjacent to the muscle section, and used to get background-subtracted values, to normalize for possible differences between sections and slides.

### Plasma glucose and insulin measurement

The concentration of insulin was measured in plasma by double-antibody immunoassay, the concentration of glucose by use of the hexokinase reaction. Measurements were carried out on Cobas c502 and e801 automated clinical chemistry analyzers respectively (Roche Diagnostics GmbH). The estimate of insulin resistance by HOMA-IR was calculated with the formula fasting serum insulin (µU/ml)x fasting plasma glucose (mmol/l)/22.5, as described (66).

### Computational proteomics

The MaxQuant software (version 1.6.15.0) was used for the analysis of raw files searching against the human Uniprot databases (UP000005640_9606 and UP000005640_9606_additional) and a common contaminants database (67). The FDR was set to 1% for peptides (minimum length of seven amino acids) and proteins and was determined by searching a reverse database. Peptide identifications by MS/MS were transferred by matching between the runs with a 0.7-min retention-time match window between single fibers, the two libraries and five technical replicates of total lysate before fractionation. Peptides with a minimum length of seven amino acids were considered for the search including N-terminal acetylation and methionine oxidation as variable modifications and cysteine carbamidomethylation as fixed modification. Enzyme specificity was set to trypsin cleaving c-terminal to arginine and lysine. A maximum of two missed cleavages was allowed. For MYH isoforms specifically, only peptides unique to each isoform were used for quantification in MaxQuant.

### Bioinformatic and statistical analysis

All analyses were performed with the Perseus software (version 1.6.14.0), part of the MaxQuant environment (68). Label-free quantification values were used throughout the analysis for protein expression, using the feature implemented in MaxQuant (67). ANOVA was performed using 0.05 FDR for truncation and 250 randomizations unless otherwise specified. Tukey's Honest Significant Difference procedure was used as post hoc test. Unequal variance was assumed between the groups and Welch t test was used for two samples comparisons with *P* < 0.05 as a threshold. Categorical annotations were supplied in the form of UniProt Keywords, Corum, KEGG, and Gene Ontology. Annotation enrichments were calculated by Fisher exact test using 0.02 Benjamini–Hochberg FDR for truncation and the Uniprot human proteome as background. Individual fibers were treated as individual samples (biological replicates) and statistics did not take the subjects of origin into account (based on Pearson correlation in Figure S1 (Supplementary Material) and see (27). For Figs3 and4, protein expression in different fiber types was converted into circles relative to the expression in type 1 fibers set at a fixed diameter. For the single fibers dataset, *n* was as follows: (i) time. BR0, *n* = 78; BR5, *n* = 75; BR10, *N* = 80. (ii) fiber type. 1-slow, *n* = 82; fast-2A, *n* = 56; fast-2X, *n* = 25; mixed1/2A, *n* = 17; mixed2A/2X, *n* = 53. For the astronaut dataset, each biopsy was measured in technical quintuplicates. ANOVA was performed separately for each astronaut and the lists of significant proteins (*P* < 0.05) were crossed for common hits only. For graphs with column scatter, *n* = 10 at three time points.

### Fiber type assignment

We used the proteomic quantification of four adult MYH isoforms to assign fiber type by MS analysis. Since different isoforms of MYH have more than 80% sequence identity; we based our quantification on the intensities of peptides unique for each isoform (69, 70). We summed the intensities of MYH7 (slow-type 1), MYH2 (fast-2A), MYH1 (fast-2X), and MYH4 (Fast-2B) and calculated for each fiber their respective amount in %. We initially split the fibers into three groups according to the majority isoform they expressed. We then calculated the median expression of the majority MYH isoform as a proxy for purity, which was 98% for MYH7/slow-1 and 78% for both MYH2/fast 2A and MYH1/fast-X (MYH4 is present only in trace amounts in humans). Approximating to lower 10%, we set a threshold of 90% for MYH7 and 70% for MYH1 and MYH2.

## Supplementary Material

pgac086_Supplemental_Files

## Data Availability

All study data are included in the article, SI Appendix, and Datasets S2–S10. The mass spectrometry proteomics data have been deposited to the ProteomeXchange Consortium via the PRIDE partner repository with the dataset identifier PXD028435.

## References

[1] Schiaffino S , Reggiani C. 2011. Fiber types in mammalian skeletal muscles. Physiol Rev. 91:1447–1531. 22013216 10.1152/physrev.00031.2010

[2] Kilroe SP , et al. 2021. Dietary protein intake does not modulate daily myofibrillar protein synthesis rates or loss of muscle mass and function during short-term immobilization in young men: a randomized controlled trial. Am J Clin Nutr. 113:548–561. 32469388 10.1093/ajcn/nqaa136

[3] Trappe TA , Burd NA, Louis ES, Lee GA, Trappe SW. 2007. Influence of concurrent exercise or nutrition countermeasures on thigh and calf muscle size and function during 60 days of bed rest in women. Acta Physiol. 191:147–159. 10.1111/j.1748-1716.2007.01728.x 17655736

[4] de Boer MD , Maganaris CN, Seynnes OR, Rennie MJ, Narici MV. 2007. Time course of muscular, neural and tendinous adaptations to 23 day unilateral lower-limb suspension in young men. J Physiol. 583:1079–1091. 17656438 10.1113/jphysiol.2007.135392 PMC2277190

[5] Rittweger J , et al. 2018; Sarcolab pilot study into skeletal muscle's adaptation to long-term spaceflight. NPJ Micrograv. 4:18. 10.1038/s41526-018-0052-1 PMC6141586 30246141

[6] Demangel R , et al. 2017. Early structural and functional signature of 3-day human skeletal muscle disuse using the dry immersion model. J Physiol. 595:4301–4315. 28326563 10.1113/JP273895 PMC5491890

[7] Brocca L , et al. 2017. FoxO-dependent atrogenes vary among catabolic conditions and play a key role in muscle atrophy induced by hindlimb suspension. J Physiol. 595:1143–1158. 27767211 10.1113/JP273097 PMC5309360

[8] Fitts RH , et al. 2010. Prolonged space flight-induced alterations in the structure and function of human skeletal muscle fibres. J Physiol. 588:3567–3592. 20660569 10.1113/jphysiol.2010.188508 PMC2988519

[9] Monti E , et al. 2021. Neuromuscular junction instability and altered intracellular calcium handling as early determinants of force loss during unloading in humans. J Physiol. 599:3037–3061. 33881176 10.1113/JP281365 PMC8359852

[10] Trappe T. 2009. Influence of aging and long-term unloading on the structure and function of human skeletal muscle. Appl Physiol Nutr Metab. 34:459–464. 19448715 10.1139/h09-041 PMC3056056

[11] Dirks ML , et al. 2016. One week of bed rest leads to substantial muscle atrophy and induces whole-body insulin resistance in the absence of skeletal muscle lipid accumulation. Diabetes. 65:2862–2875. 27358494 10.2337/db15-1661

[12] Memme JM , Slavin M, Moradi N, Hood DA. 2021. Mitochondrial bioenergetics and turnover during chronic muscle disuse. Int J Mol Sci. 22:5179. 34068411 10.3390/ijms22105179 PMC8153634

[13] Hawley JA , Hargreaves M, Joyner MJ, Zierath JR. 2014. Integrative biology of exercise. Cell. 159:738–749. 25417152 10.1016/j.cell.2014.10.029

[14] English KL , et al. 2020. High intensity training during spaceflight: results from the NASA Sprint Study. NPJ Micrograv. 6:21. 10.1038/s41526-020-00111-x PMC7434884 32864428

[15] Petersen N , et al. 2016. Exercise in space: the European Space Agency approach to in-flight exercise countermeasures for long-duration missions on ISS. Extrem Physiol Med. 5:9. 27489615 10.1186/s13728-016-0050-4 PMC4971634

[16] Afshinnekoo E , et al. 2020. Fundamental biological features of spaceflight: advancing the field to enable deep-space exploration. Cell. 183:1162–1184. 33242416 10.1016/j.cell.2020.10.050 PMC8441988

[17] da Silveira WA , et al. 2020. Comprehensive multi-omics analysis reveals mitochondrial stress as a central biological hub for spaceflight impact. Cell. 183:1185–1201 e1120. 33242417 10.1016/j.cell.2020.11.002 PMC7870178

[18] Fitts RH , et al. 2013. Effects of prolonged space flight on human skeletal muscle enzyme and substrate profiles. J Appl Physiol. 115:667–679. 23766501 10.1152/japplphysiol.00489.2013

[19] Garrett-Bakelman FE , et al. 2019. The NASA twins study: a multidimensional analysis of a year-long human spaceflight. Science. 364:eaau8650. 30975860 10.1126/science.aau8650 PMC7580864

[20] Patel ZS , et al. 2020. Red risks for a journey to the red planet: the highest priority human health risks for a mission to Mars. NPJ Micrograv. 6:33. 10.1038/s41526-020-00124-6 PMC7645687 33298950

[21] Mahmassani ZS , et al. 2019. Age-dependent skeletal muscle transcriptome response to bed rest-induced atrophy. J Appl Physiol. 126:894–902. 30605403 10.1152/japplphysiol.00811.2018 PMC6485685

[22] Nay K , et al. 2020. Simulated microgravity disturbs iron metabolism and distribution in humans: lessons from dry immersion, an innovative ground-based human model. FASEB J. 34:14920–14929. 32918768 10.1096/fj.202001199RR

[23] Hong X , et al. 2021. Effects of spaceflight aboard the International Space Station on mouse estrous cycle and ovarian gene expression. NPJ Micrograv. 7:11. 10.1038/s41526-021-00139-7 PMC7954810 33712627

[24] Thiel CS , et al. 2018. Rapid coupling between gravitational forces and the transcriptome in human myelomonocytic U937 cells. Sci Rep. 8:13267. 30185876 10.1038/s41598-018-31596-y PMC6125427

[25] Deshmukh AS , et al. 2015. Deep proteomics of mouse skeletal muscle enables quantitation of protein isoforms, metabolic pathways, and transcription factors. Mol Cell Proteomics. 14:841–853. 25616865 10.1074/mcp.M114.044222 PMC4390264

[26] Deshmukh AS , et al. 2021. Deep muscle-proteomic analysis of freeze-dried human muscle biopsies reveals fiber type-specific adaptations to exercise training. Nat Commun. 12:304. 33436631 10.1038/s41467-020-20556-8 PMC7803955

[27] Murgia M , et al. 2017. Single muscle fiber proteomics reveals fiber-type-specific features of human muscle aging. Cell Rep. 19:2396–2409. 28614723 10.1016/j.celrep.2017.05.054

[28] Vettor R , et al. 2009. The origin of intermuscular adipose tissue and its pathophysiological implications. Am J Physiol Endocrinol Metab. 297:E987–E998. 19738037 10.1152/ajpendo.00229.2009

[29] Brunner AD , et al. 2022. Ultra-high sensitivity mass spectrometry quantifies single-cell proteome changes upon perturbation. Mol Syst Biol. 18:e10798. 35226415 10.15252/msb.202110798 PMC8884154

[30] Clement GR , et al. 2020. Challenges to the central nervous system during human spaceflight missions to Mars. J Neurophysiol. 123:2037–2063. 32292116 10.1152/jn.00476.2019

[31] Gallo C , Ridolfi L, Scarsoglio S. 2020. Cardiovascular deconditioning during long-term spaceflight through multiscale modeling. NPJ Micrograv. 6:27. 10.1038/s41526-020-00117-5 PMC7529778 33083524

[32] Vico L , Hargens A. 2018. Skeletal changes during and after spaceflight. Nat Rev Rheumatol. 14:229–245. 29559713 10.1038/nrrheum.2018.37

[33] Schiaffino S , Reggiani C, Murgia M. 2020. Fiber type diversity in skeletal muscle explored by mass spectrometry-based single fiber proteomics. Histol Histopathol. 35:239–246. 31612964 10.14670/HH-18-170

[34] Henderson CA , Gomez CG, Novak SM, Mi-Mi L, Gregorio CC. 2017. Overview of the muscle cytoskeleton. Compr Physiol. 7:891–944. 28640448 10.1002/cphy.c160033 PMC5890934

[35] Rybakova IN , Patel JR, Ervasti JM. 2000. The dystrophin complex forms a mechanically strong link between the sarcolemma and costameric actin. J Cell Biol. 150:1209–1214. 10974007 10.1083/jcb.150.5.1209 PMC2175263

[36] Cohen S , Lee D, Zhai B, Gygi SP, Goldberg AL. 2014. Trim32 reduces PI3K-Akt-FoxO signaling in muscle atrophy by promoting plakoglobin-PI3K dissociation. J Cell Biol. 204:747–758. 24567360 10.1083/jcb.201304167 PMC3941042

[37] Mutlak YE , et al. 2020. A signaling hub of insulin receptor, dystrophin glycoprotein complex and plakoglobin regulates muscle size. Nat Commun. 11:1381. 32170063 10.1038/s41467-020-14895-9 PMC7070008

[38] Park MH. 2006. The post-translational synthesis of a polyamine-derived amino acid, hypusine, in the eukaryotic translation initiation factor 5A (eIF5A). J Biochem. 139:161–169. 16452303 10.1093/jb/mvj034 PMC2494880

[39] Porter MM , Vandervoort AA, Lexell J. 1995. Aging of human muscle: structure, function and adaptability. Scand J Med Sci Sports. 5:129–142. 7552755 10.1111/j.1600-0838.1995.tb00026.x

[40] Favaro G , et al. 2019. DRP1-mediated mitochondrial shape controls calcium homeostasis and muscle mass. Nat Commun. 10:2576. 31189900 10.1038/s41467-019-10226-9 PMC6561930

[41] Tezze C , et al. 2017. Age-associated loss of OPA1 in muscle impacts muscle mass, metabolic homeostasis, systemic inflammation, and epithelial senescence. Cell Metab. 25:1374–1389 e1376. 28552492 10.1016/j.cmet.2017.04.021 PMC5462533

[42] Purves-Smith FM , Sgarioto N, Hepple RT. 2014. Fiber typing in aging muscle. Exerc Sport Sci Rev. 42:45–52. 24508741 10.1249/JES.0000000000000012

[43] Tsika RW , Herrick RE, Baldwin KM. 1987. Time course adaptations in rat skeletal muscle isomyosins during compensatory growth and regression. J Appl Physiol. 63:2111–2121. 2961724 10.1152/jappl.1987.63.5.2111

[44] Boppart MD , Mahmassani ZS. 2019. Integrin signaling: linking mechanical stimulation to skeletal muscle hypertrophy. Am J Physiol Cell Physiol. 317:C629–C641. 31314586 10.1152/ajpcell.00009.2019 PMC6850995

[45] Thot GK , et al. 2021. Effects of long-term immobilisation on endomysium of the soleus muscle in humans. Exp Physiol. 106:2038–2045. 34387385 10.1113/EP089734

[46] Nagarajan A , et al. 2016. MARCH1 regulates insulin sensitivity by controlling cell surface insulin receptor levels. Nat Commun. 7:12639. 27577745 10.1038/ncomms12639 PMC5013666

[47] Hughson RL , et al. 2016. Increased postflight carotid artery stiffness and inflight insulin resistance resulting from 6-mo spaceflight in male and female astronauts. Am J Physiol Heart Circ Physiol. 310:H628–H638. 26747504 10.1152/ajpheart.00802.2015

[48] Brocca L , et al. 2012. The time course of the adaptations of human muscle proteome to bed rest and the underlying mechanisms. J Physiol. 590:5211–5230. 22848045 10.1113/jphysiol.2012.240267 PMC3497573

[49] Dillon EL , et al. 2019. Proteomic investigation of human skeletal muscle before and after 70 days of head down bed rest with or without exercise and testosterone countermeasures. PLoS ONE. 14:e0217690. 31194764 10.1371/journal.pone.0217690 PMC6563988

[50] Moriggi M , et al. 2010. Long term bed rest with and without vibration exercise countermeasures: effects on human muscle protein dysregulation. Proteomics. 10:3756–3774. 20957755 10.1002/pmic.200900817

[51] Fluck M , et al. 2020. Down-regulation of mitochondrial metabolism after tendon release primes lipid accumulation in rotator cuff muscle. Am J Pathol. 190:1513–1529. 32305353 10.1016/j.ajpath.2020.03.019

[52] Fernandez-Gonzalo R , et al. 2020. Three months of bed rest induce a residual transcriptomic signature resilient to resistance exercise countermeasures. FASEB J. 34:7958–7969. 32293758 10.1096/fj.201902976R

[53] Balagopal P , Ljungqvist O, Nair KS. 1997. Skeletal muscle myosin heavy-chain synthesis rate in healthy humans. Am J Physiol. 272:E45–E50. 9038850 10.1152/ajpendo.1997.272.1.E45

[54] Alibegovic AC , et al. 2010. Insulin resistance induced by physical inactivity is associated with multiple transcriptional changes in skeletal muscle in young men. Am J Physiol Endocrinol Metab. 299:E752–E763. 20739510 10.1152/ajpendo.00590.2009

[55] Lin KH , et al. 2021. A deep analysis of the proteomic and phosphoproteomic alterations that occur in skeletal muscle after the onset of immobilization. J Physiol. 599:2887–2906. 33873245 10.1113/JP281071 PMC8353513

[56] Puleston DJ , et al. 2019. Polyamines and eIF5A hypusination modulate mitochondrial respiration and macrophage activation. Cell Metab. 30:352–363 e358. 31130465 10.1016/j.cmet.2019.05.003 PMC6688828

[57] Zhang H , et al. 2019. Polyamines control eIF5A hypusination, TFEB translation, and autophagy to reverse B cell senescence. Mol Cell. 76:110–125 e119. 31474573 10.1016/j.molcel.2019.08.005 PMC6863385

[58] Romanello V , Sandri M. 2021. The connection between the dynamic remodeling of the mitochondrial network and the regulation of muscle mass. Cell Mol Life Sci. 78:1305–1328. 33078210 10.1007/s00018-020-03662-0 PMC7904552

[59] Bell MB , Bush Z, McGinnis GR, Rowe GC. 2019. Adult skeletal muscle deletion of Mitofusin 1 and 2 impedes exercise performance and training capacity. J Appl Physiol. 126:341–353. 30260752 10.1152/japplphysiol.00719.2018 PMC6397407

[60] Konig T , et al. 2016. The m-AAA protease associated with neurodegeneration limits MCU activity in mitochondria. Mol Cell. 64:148–162. 27642048 10.1016/j.molcel.2016.08.020

[61] Indo HP , et al. 2016. Changes in mitochondrial homeostasis and redox status in astronauts following long stays in space. Sci Rep. 6:39015. 27982062 10.1038/srep39015 PMC5159838

[62] Cucinotta FA. 2014. Space radiation risks for astronauts on multiple International Space Station missions. PLoS ONE. 9:e96099. 24759903 10.1371/journal.pone.0096099 PMC3997516

[63] Fix DK , Hardee JP, Bateman TA, Carson JA. 2016. Effect of irradiation on Akt signaling in atrophying skeletal muscle. J Appl Physiol. 121:917–924. 27562841 10.1152/japplphysiol.00218.2016 PMC5142305

[64] Cadena SM , et al. 2019. Skeletal muscle in MuRF1 null mice is not spared in low-gravity conditions, indicating atrophy proceeds by unique mechanisms in space. Sci Rep. 9:9397. 31253821 10.1038/s41598-019-45821-9 PMC6599046

[65] Kulak NA , Geyer PE, Mann M. 2017. Loss-less nano-fractionator for high sensitivity, high coverage proteomics. Mol Cell Proteomics. 16:694–705. 28126900 10.1074/mcp.O116.065136 PMC5383787

[66] Matthews DR , et al. 1985. Homeostasis model assessment: insulin resistance and beta-cell function from fasting plasma glucose and insulin concentrations in man. Diabetologia. 28:412–419. 3899825 10.1007/BF00280883

[67] Cox J , et al. 2014. Accurate proteome-wide label-free quantification by delayed normalization and maximal peptide ratio extraction, termed MaxLFQ. Mol Cell Proteomics. 13:2513–2526. 24942700 10.1074/mcp.M113.031591 PMC4159666

[68] Tyanova S , et al. 2016. The Perseus computational platform for comprehensive analysis of (prote)omics data. Nat Methods. 13:731–740. 27348712 10.1038/nmeth.3901

[69] Drexler HC , et al. 2012. On marathons and sprints: an integrated quantitative proteomics and transcriptomics analysis of differences between slow and fast muscle fibers. Mol Cell Proteomics. 11:M111 010801. 10.1074/mcp.M111.010801 PMC3433927 22210690

[70] Fraterman S , Zeiger U, Khurana TS, Wilm M, Rubinstein NA. 2007. Quantitative proteomics profiling of sarcomere associated proteins in limb and extraocular muscle allotypes. Mol Cell Proteomics. 6:728–737. 17229715 10.1074/mcp.M600345-MCP200

