# Supplementary material for: Signatures of muscle disuse in spaceflight and bed rest revealed by single muscle fiber proteomics
Source: PNAS Nexus. 2022 Jun 11;1(3):pgac086. doi: 10.1093/pnasnexus/pgac086 (PMC9896895; doi:10.1093/pnasnexus/pgac086)
Supplement: pgac086_Supplemental_Files [file pgac086_supplemental_files.zip › PNASNEXUS-PNASNEXUS-2022-00455-T-s01.docx]

**
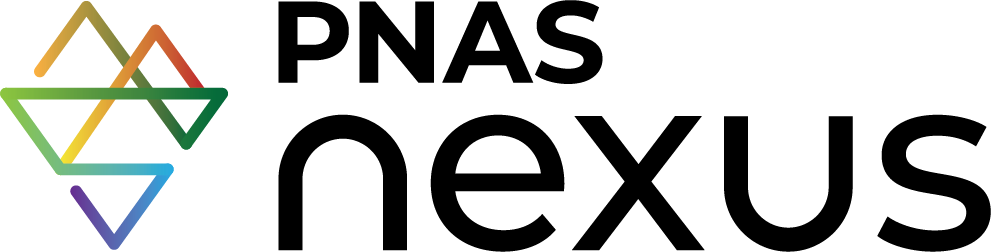
**

**Supplementary Information for**

Signatures of muscle disuse in spaceflight and bed rest revealed by single muscle fiber proteomics.

Marta Murgia ^1,2^*, Stefano Ciciliot ^3,4^, Nagarjuna Nagaraj ^5,^ Carlo Reggiani ^1,6^, Stefano Schiaffino ^3^, Martino V Franchi ^1^, Rado Pišot ^6^, Boštjan Šimunič ^6^, Luana Toniolo ^1^, Bert Blaauw ^1,3^, Marco Sandri ^1,3^, Gianni Biolo ^7^, Martin Flück ^8^, Marco V Narici ^1,6, 9^ and Matthias Mann ^2,10^*.

*Correspondence: Marta Murgia and Matthias Mann.

**Email:**  mmurgia@biochem.mpg.de, mmann@biochem.mpg.de

**This PDF file includes:**

Supplementary text

Figures S1 to S4

Table S1

Legends for Datasets S2 to S10

**Other supplementary materials for this manuscript include the following:**

Datasets S2 to S10


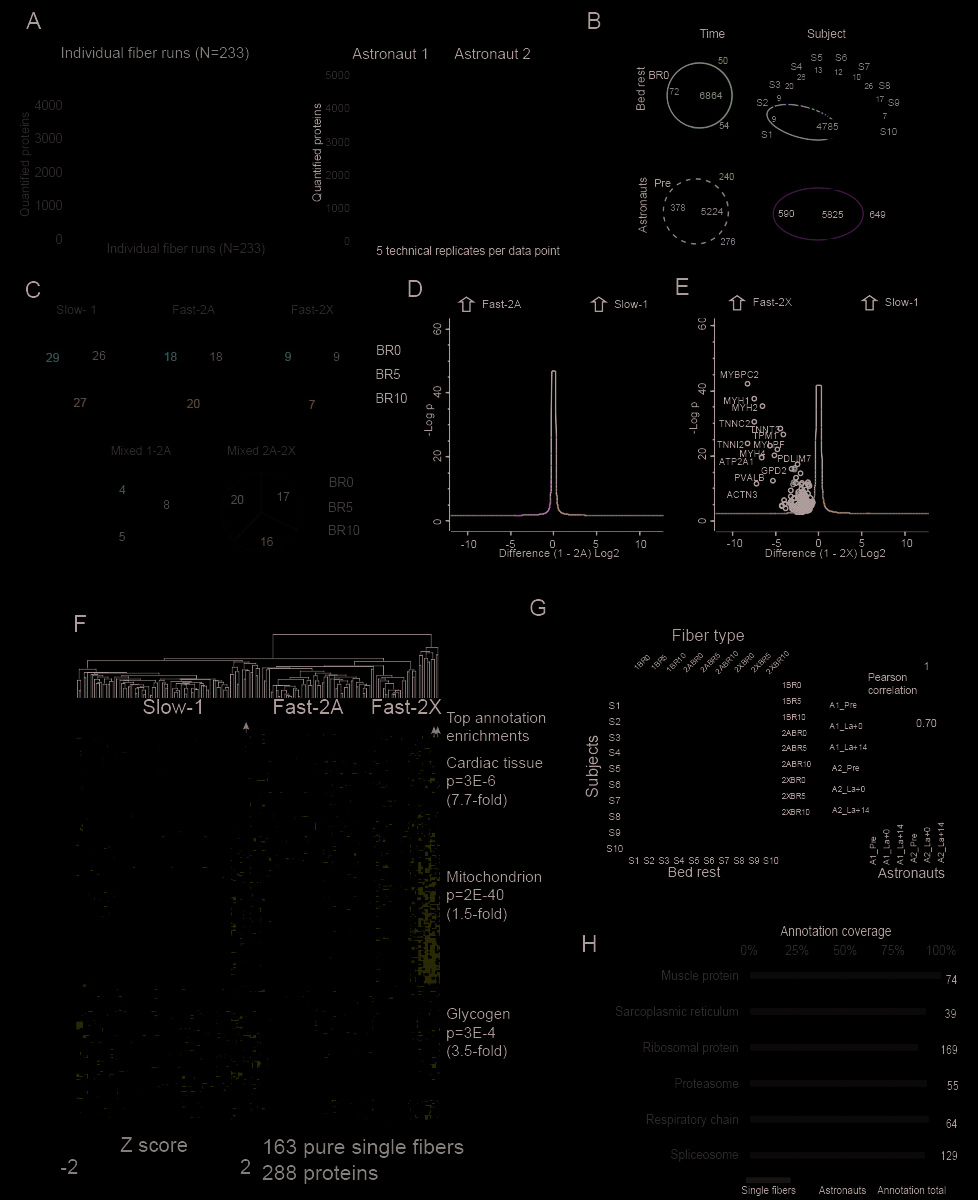


Fig. S1. Proteomic features of the dataset and fiber type phenotype (related to Figure 1)

(A) Number of proteins quantified by shotgun proteomics in all 233 single fibers from ten subjects at three time points of bed rest and in two astronauts at three time points before and after a six months mission on the International Space Station (technical quintuplicates). (B) Venn diagrams showing the number of common and exclusive proteins in the bed rest (three time points and ten study subjects) and astronaut datasets (three time points and two study subjects). The total number of proteins quantified in all samples of each subject (single fibers for bed rest subjects, replicates for astronauts) were used for the diagrams. (C) Number of single fibers at the three BR times, calculated for three pure and two mixed fiber types. (D) Proteins with significantly different expression between pure Slow-1 (blue) and Fast-2A fibers (green). N=86 and 52 single fibers respectively. Data were filtered for 10 valid values in at least one group (5588 protein groups). (E) Proteins with significantly different expression between pure Slow-1 (blue) and Fast-2X fibers (Black). N=86 and 25 single fibers respectively. Data were filtered for 10 valid values in at least one group (5588 protein groups). (F) Unsupervised hierarchical clustering of pure fiber types based on proteins with significant expression difference between at least 2 out of three groups (ANOVA). Only three exceptions were observed, one fast-2A fibers grouping with slow-1 and two with fast-2X fibers (arrows). Fiber type is indicated by the color map on top (slow-1, blue, fast-2A, green, fast-2X dark grey). N=163 pure fibers, 232 significant proteins. Top annotation enrichments in clusters are labeled on the right, with p value and fold enrichment. Cardiac tissue, 6 of 7 proteins in cluster; glycogen storage disease 7 of 8; mitochondrion 88 of 98. (G) Left panel, matrix of correlations between label-free quantification (LFQ) intensities of ten subjects, median of all fibers per subject (left) and three pure fiber types, median of three bed rest (BR) times per fiber type (right). Right panel, same analysis for astronauts. Median of five technical replicates per data point. Pearson correlation coefficients performed at the whole proteome level for each comparison are indicated by a color scale, top right. (H) Protein annotation coverage in muscle fibers. Each bar represents a selected category of Keyword annotations. The total number of protein groups in the human proteome with the same annotation represents 100%. Proteins quantified in muscle fibers correspond to the orange bar segments.


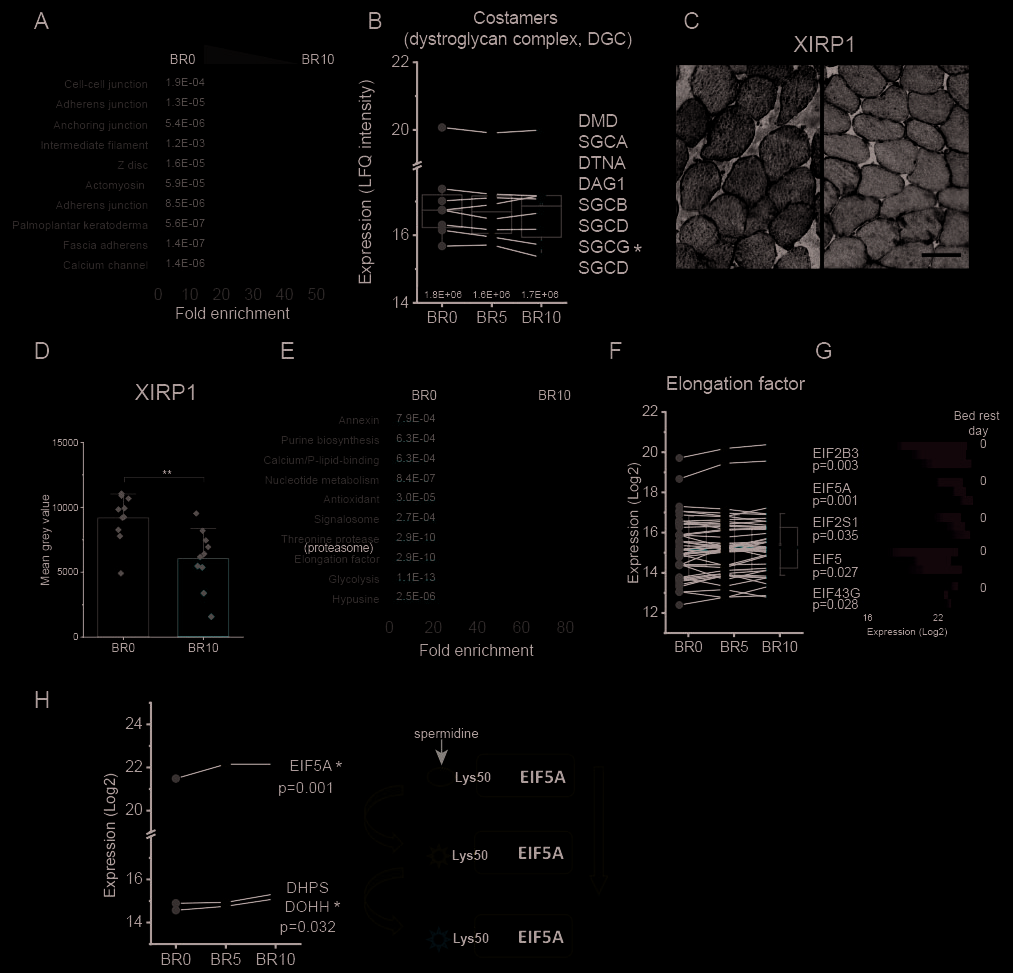


Fig. S2. Effects of muscle disuse on sarcomere and excitation-contraction machinery (related to Figure 2)

(A) Annotation enrichment in proteins with significantly higher expression pre-bed rest (BR0) compared to BR10. Bar graphs show fold enrichment in the indicated annotation corresponding p value (Fisher exact test). (B) Trendlines with half boxplots of the expression of proteins of the dystroglycan complex (DGC). Box shows median, 75th and 25th percentile, whiskers show SD. Protein list is shown on the right, ordered by expression at BR10 in decreasing order. Only gamma sarcoglycan (SGCG) shows statistically significant expression difference in fibers at BR0 compared to BR10 (*p<0.05). The summed intensity of the proteins at each time point is shown above the x axis. (C) Immunofluorescence analysis of XIRP1 at BR0 and BR10 in muscle biopsies of bed rest subjects. Representative images of the staining at BR0 and BR10 of the same subject. Brightness was digitally enhanced by an identical factor (150) for the two images. (D) Median grey value in the stained biopsy of all subjects (N=10, >50 fibers/subject, T-test). (E) Annotation enrichment in proteins with significantly higher expression at BR10 compared to pre-bed rest (BR0). Bar graphs show fold enrichment in the indicated annotation corresponding p value (Fisher exact test). (F)Trendlines with half boxplots of the expression of proteins involved in translation initiation (Keywords annotation Initiation factor). See panel B. (G) Heatmap showing the expression in all fibers of five translation initiation factors with significant increase in expression upon unloading. Protein expression (LFQ intensity, Log2, color scale at bottom) is split into horizontal blocks, showing all fibers at each time of bed rest, as indicated on the right. BR0, 78 fibers, BR5 75, BR10 80. P value (T-test, BR10 vs BR0) is reported on the left. (H) Boxplot with trendlines of the expression of the translational initiation/elongation factor EIF5A and of two enzymes responsible for the conversion of lysine-50 to hypusine. Box shows median, 75th and 25th percentile. Each protein is labelled on the right, with corresponding p value for significant ones (T-test, BR10 vs BR0). On the right, schematic representation of the hypusination pathway.


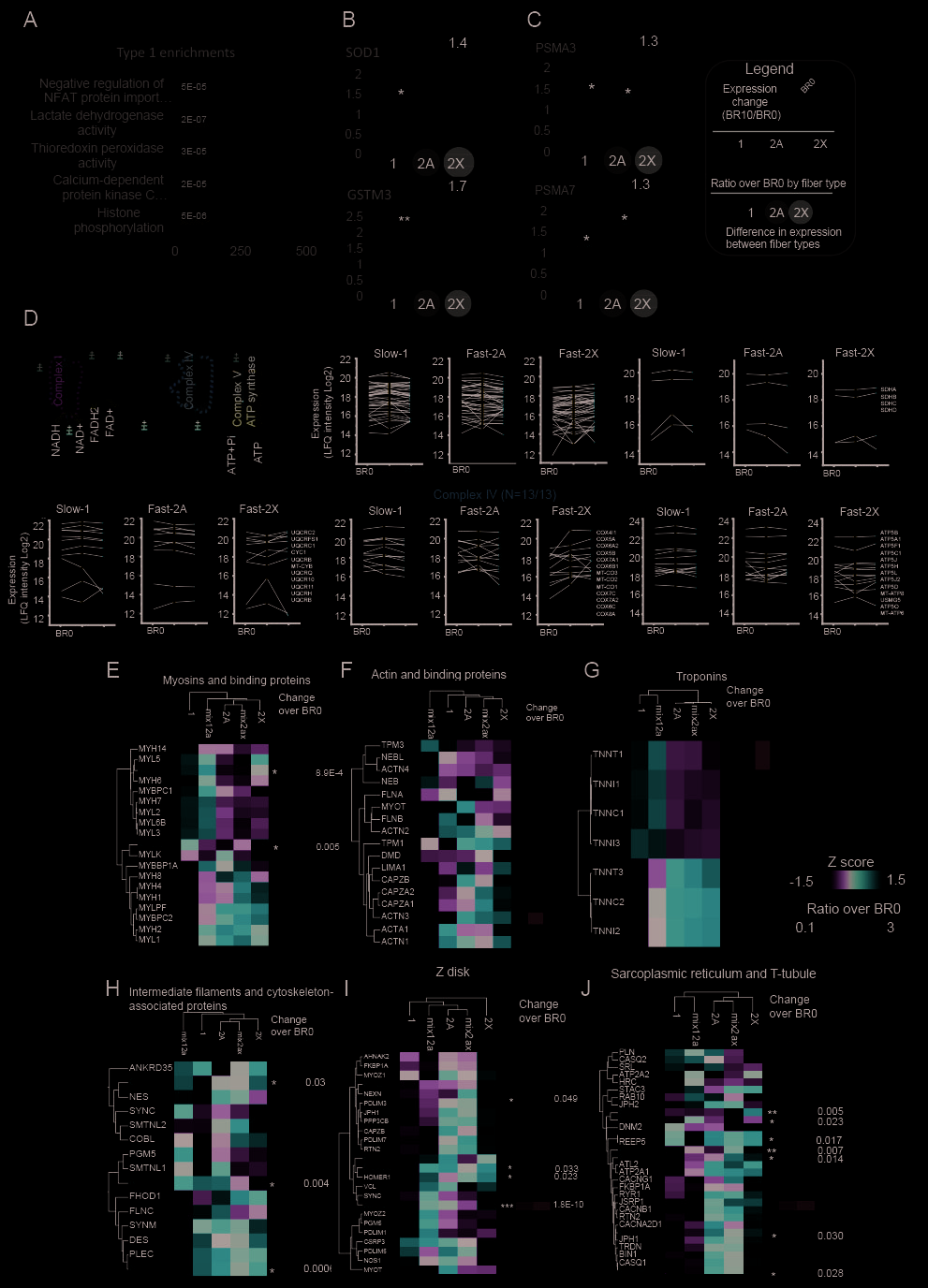
 Fig. S3. Changes in muscle fiber adhesion structures during bed rest (related to Figure 3)

Annotation enrichment in proteins with significantly higher expression at BR10 compared to BR0 in slow-1 fibers only. Bar graphs show fold enrichment in the indicated annotation corresponding p value (Fisher exact test). (B) Fiber type-resolved effects of muscle unloading in two redox proteins upregulated by unloading only in type1-slow fibers. The ratio between expression at BR10 and BR0 in all fibers (top graph), the ratio in individual pure fiber types (middle graph) and the relative expression in different fiber type (bottom circles) is shown as depicted in the legend. BR0, grey bars: BR5, blue bars: BR10, red bars. T-test (*p<0.05). See detailed legend on the right of panel C. (C) Fiber type-resolved effects of muscle unloading in subunits of the proteasome upregulated by unloading only in slow-1 and fast-2A, but not fast-2X. (D) Schematic representation of the five respiratory chain complexes and proton translocation across the inner mitochondrial membrane and fiber type-resolved profile plots showing the expression of each mitochondrial respiratory chain complex (labelled on top of each graph. For all complexes except I, proteins are listed on the right in descending intensity order (as in Fast-2X BR10). (E) Pure and mixed-type fiber type-resolved heat maps of the expression (LFQ intensity, Z-scored, see scale at figure bottom) of myosins and myosin-binding proteins. In this panel only, protein quantification by unique peptides only due to high subunit homology of MYHs. On the right, heat map of the expression ratio between bed rest day 10 (BR10) and pre-bed rest (BR0) in all fibers regardless of type, color scale at figure bottom. Proteins (gene names) with significant expression changes are marked in red and indicated with an asterisk and corresponding P value on the right. T-test, N=78 fibers at BR0 and =80 at BR10. (F) Pure- and mixed-type fiber type-resolved heat maps of the expression of sarcomeric actin (ACTA1) and various actin-binding proteins (also binding other actin isoforms). Standard protein quantification with unique and razor peptides. See A for description. (G) Pure- and mixed-type fiber type-resolved heat maps of the expression of troponins. Standard protein quantification with unique and razor peptides. See A for description. (H) Pure- and mixed-type fiber type-resolved heat maps of the expression of intermediate filaments proteins. Standard protein quantification with unique and razor peptides. See A for description. (I) Pure- and mixed-type fiber type-resolved heat maps of the expression of Z disk proteins. See A for description. (J) Pure- and mixed-type fiber type-resolved heat maps of the expression of sarcoplasmic reticulum (SR) and T-tubule proteins. See A for description.


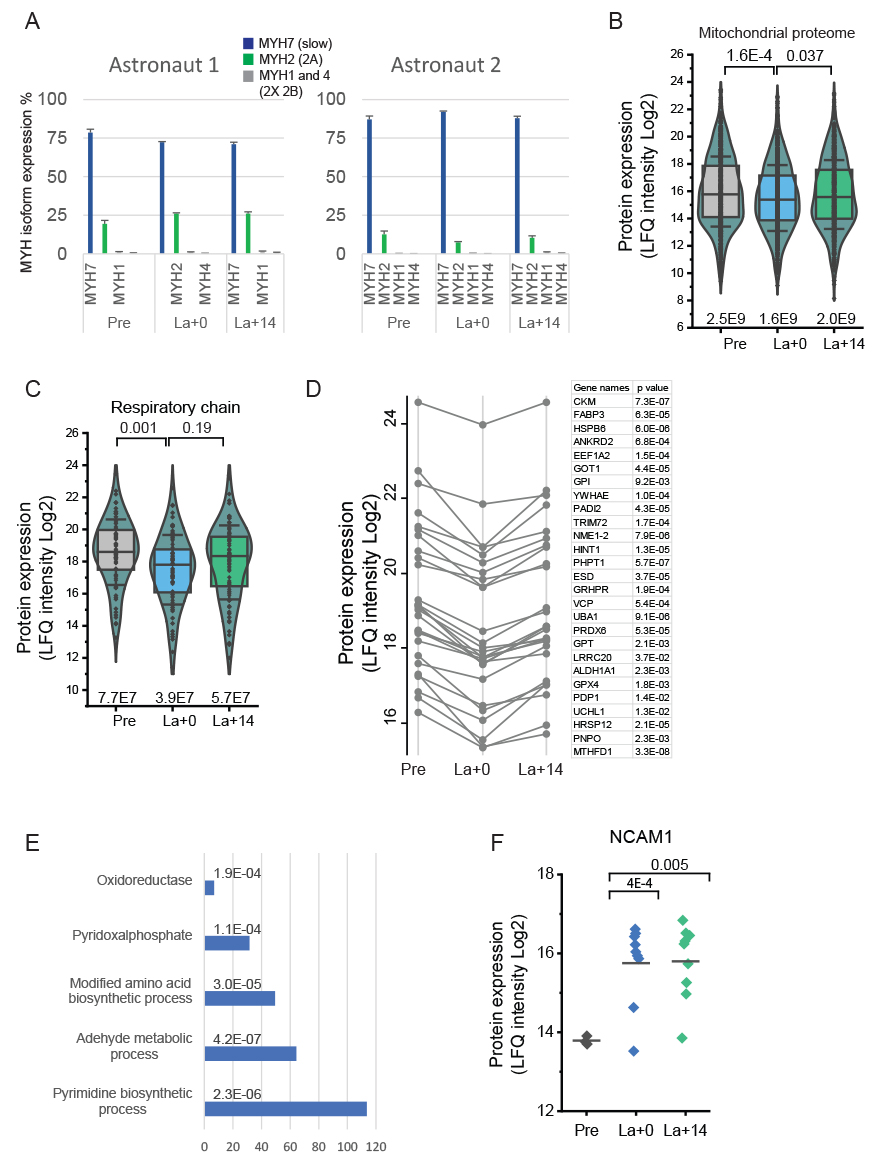


Fig. S4. Loading-dependent protein expression changes in astronauts and bed rest

(A) Expression of MYH isoforms at different phases of the mission for each astronaut separately. Bars show median expression (percent of the four adult MYH isoforms), error bars standard deviation. N=5 technical replicates. (B) Violin plot of the muscle mitochondrial proteome of astronauts before mission (Pre), on the day of landing (La+0) and after 14 days of normal activity on Earth (La+14). The area encompasses all data points at each time. Box shows median, 25th and 75th percentile, bars SD. Summed intensity of all mitochondrial proteins is shown on top of the x axis. P value of T-test between time points on top. Based on 862 proteins. (C) Same analysis for the respiratory chain of astronauts. Based on 82 proteins. (D) Profile plots of the expression of common proteins upregulated during bed rest and spaceflight. Proteins are listed on the right, ordered by decreasing expression at La+14, with value of T-test (Pre vs La+0, N=10, 2 astronauts, 5 technical replicates,). (E)Top 5 annotation enrichments, with p value, in proteins upregulated by both bed rest and spaceflight. (F) Expression of neural cell adhesion molecule 1 (NCAM1) in skeletal muscle before (Pre), on the day of landing (La+0) and 14 days after landing (La+14). N= 10, 2 astronauts, 5 technical replicates.

Table S1. Biometric data and HOMA-IR index of the ten subjects recruited for the bed rest study.

| **Subject** | **Age (years)** | **Height (cm)** | **Weight BR0 (Kg)** | **Weight BR10**  **(Kg)** | **HOMA score BR0** | **HOMA score BR10** |
| --- | --- | --- | --- | --- | --- | --- |
| S1 | 33 | 185.5 | 87.6 | 85.4 | 1.06 | 1.27 |
| S2 | 21 | 181.5 | 69.9 | 70.4 | 1.40 | 1.21 |
| S3 | 19 | 178 | 67.5 | 66.8 | 1.47 | 2.18 |
| S4 | 21 | 182.5 | 73.4 | 69.9 | 1.29 | 0.90 |
| S5 | 18 | 189.1 | 96.8 | 92.7 | 1.85 | 2.83 |
| S6 | 25 | 177.8 | 77.5 | 78.5 | 0.97 | 1.41 |
| S7 | 28 | 178.5 | 88.2 | 87.3 | 3.11 | 3.31 |
| S8 | 24 | 181.7 | 71 | 69.8 | 0.96 | 3.14 |
| S9 | 21 | 181 | 75.5 | 72.5 | 1.28 | 1.52 |
| S10 | 20 | 176.2 | 67.7 | 66.3 | 0.98 | 0.98 |

E

BR0, before bed rest; BR10, bed rest day 10. HOMA-IR index, HOmeostatic Model Assessment for Insulin Resistance was calculated from fasting plasma insulin in mIU/L and glucose in mg/dL.

TableS2

Label free quantification (LFQ) intensity of the proteome of 233 single fibers from 10 subjects before and during bed rest. (Related to Figure 1)

**TableS3.**

Label free quantification (LFQ) intensity of the muscle proteome of two astronauts before and after a mission on the International Space Station. (Related to Figure 1)

**TableS4.**

Main and other isoform composition of individual fibers. (Related to Figure 1)

**TableS5**.

Proteins with significantly different expression between slow-1 and fast-2A fibers (Related to Figure S1)

**TableS6.**

Proteins with significantly different expression between slow-1 and fast-2X fibers (Related to Figure S1)

**TableS7**.

Proteins with significantly different expression between at least two of three bed rest times (Related to Figure S2, S3, S4)

**TableS8.**

Proteins significantly regulated during bed rest in each fiber type (Related to Figure 3)

**TableS9.**

Proteins with significantly different expression between at least two of three times of mission (Pre, day of landing La+0, 14 days after landing La+14) in astronauts (Related to Figure 4 and S4)

**TableS10.**

Mitochondrial proteins with significantly different expression between different phases of the mission (Pre, day of landing La+0, 14 days after landing La+14) in astronauts
